# Identification and Development of Therapeutics for COVID-19

**Published:** 2021-03-03

**Authors:** Halie M. Rando, Nils Wellhausen, Soumita Ghosh, Alexandra J. Lee, Anna Ada Dattoli, Fengling Hu, James Brian Byrd, Diane N. Rafizadeh, Yanjun Qi, Yuchen Sun, Jeffrey M. Field, Marouen Ben Guebila, Nafisa M. Jadavji, Ronan Lordan, Ashwin N. Skelly, Bharath Ramsundar, Christian Brueffer, Jinhui Wang, Rishi Raj Goel, YoSon Park, COVID-19 Review Consortium, Simina M. Boca, Anthony Gitter, Casey S. Greene

**Affiliations:** Department of Systems Pharmacology and Translational Therapeutics, University of Pennsylvania, Philadelphia, Pennsylvania, United States of America; Department of Biochemistry and Molecular Genetics, University of Colorado School of Medicine, Aurora, Colorado, United States of America; Center for Health AI, University of Colorado School of Medicine, Aurora, Colorado, United States of America · Funded by the Gordon and Betty Moore Foundation (GBMF 4552); Department of Systems Pharmacology and Translational Therapeutics, University of Pennsylvania, Philadelphia, Pennsylvania, United States of America; Institute of Translational Medicine and Therapeutics, Perelman School of Medicine, University of Pennsylvania, Philadelphia, Pennsylvania, United States of America; Department of Systems Pharmacology and Translational Therapeutics, University of Pennsylvania, Philadelphia, Pennsylvania, United States of America · Funded by the Gordon and Betty Moore Foundation (GBMF 4552); Department of Systems Pharmacology & Translational Therapeutics, Perelman School of Medicine, University of Pennsylvania, Philadelphia, PA 19104, USA; Department of Biostatistics, Epidemiology and Informatics, University of Pennsylvania, Philadelphia, Pennsylvania, United States of America; University of Michigan School of Medicine, Ann Arbor, Michigan, United States of America · Funded by NIH K23HL128909; FastGrants; Perelman School of Medicine, University of Pennsylvania, Philadelphia, Pennsylvania, United States of America; Department of Chemistry, University of Pennsylvania, Philadelphia, Pennsylvania, United States of America · Funded by NIH Medical Scientist Training Program T32 GM07170; Department of Computer Science, University of Virginia, Charlottesville, VA, United States of America; Department of Computer Science, University of Virginia, Charlottesville, VA, United States of America; Department of Pharmacology, Perelman School of Medicine, University of Pennsylvania, Philadelphia, PA 19104, USA; Department of Biostatistics, Harvard School of Public Health, Boston, Massachusetts, United States of America; Biomedical Science, Midwestern University, Glendale, AZ, United States of America; Department of Neuroscience, Carleton University, Ottawa, Ontario, Canada · Funded by the American Heart Association (20AIREA35050015); Institute for Translational Medicine and Therapeutics, Perelman School of Medicine, University of Pennsylvania, Philadelphia, PA 19104-5158, USA; Perelman School of Medicine, University of Pennsylvania, Philadelphia, Pennsylvania, United States of America; Institute for Immunology, University of Pennsylvania Perelman School of Medicine, Philadelphia, United States of America · Funded by NIH Medical Scientist Training Program T32 GM07170; The DeepChem Project, https://deepchem.io/; Department of Clinical Sciences, Lund University, Lund, Sweden; Perelman School of Medicine, University of Pennsylvania, Philadelphia, Pennsylvania, United States of America; Institute for Immunology, University of Pennsylvania, Philadelphia, PA, United States of America; Department of Systems Pharmacology and Translational Therapeutics, University of Pennsylvania, Philadelphia, Pennsylvania, United States of America · Funded by NHGRI R01 HG10067; Innovation Center for Biomedical Informatics, Georgetown University Medical Center, Washington, District of Columbia, United States of America; Department of Biostatistics and Medical Informatics, University of Wisconsin-Madison, Madison, Wisconsin, United States of America; Morgridge Institute for Research, Madison, Wisconsin, United States of America · Funded by John W. and Jeanne M. Rowe Center for Research in Virology; Department of Systems Pharmacology and Translational Therapeutics, University of Pennsylvania, Philadelphia, Pennsylvania, United States of America; Childhood Cancer Data Lab, Alex’s Lemonade Stand Foundation, Philadelphia, Pennsylvania, United States of America; Department of Biochemistry and Molecular Genetics, University of Colorado School of Medicine, Aurora, Colorado, United States of America; Center for Health AI, University of Colorado School of Medicine, Aurora, Colorado, United States of America · Funded by the Gordon and Betty Moore Foundation (GBMF 4552); the National Human Genome Research Institute (R01 HG010067)

## Abstract

After emerging in China in late 2019, the novel *Severe acute respiratory syndrome-like coronavirus 2* (SARS-CoV-2) spread worldwide and as of early 2021, continues to significantly impact most countries. Only a small number of coronaviruses are known to infect humans, and only two are associated with the severe outcomes associated with SARS-CoV-2: *Severe acute respiratory syndrome-related coronavirus*, a closely related species of SARS-CoV-2 that emerged in 2002, and *Middle East respiratory syndrome-related coronavirus*, which emerged in 2012. Both of these previous epidemics were controlled fairly rapidly through public health measures, and no vaccines or robust therapeutic interventions were identified. However, previous insights into the immune response to coronaviruses gained during the outbreaks of severe acute respiratory syndrome (SARS) and Middle East respiratory syndrome (MERS) have proved beneficial to identifying approaches to the treatment and prophylaxis of novel coronavirus disease 2019 (COVID-19). A number of potential therapeutics against SARS-CoV-2 and the resultant COVID-19 illness were rapidly identified, leading to a large number of clinical trials investigating a variety of possible therapeutic approaches being initiated early on in the pandemic. As a result, a small number of therapeutics have already been authorized by regulatory agencies such as the Food and Drug Administration (FDA) in the United States, and many other therapeutics remain under investigation. Here, we describe a range of approaches for the treatment of COVID-19, along with their proposed mechanisms of action and the current status of clinical investigation into each candidate. The status of these investigations will continue to evolve, and this review will be updated as progress is made.

## Introduction

**Figure 1:**
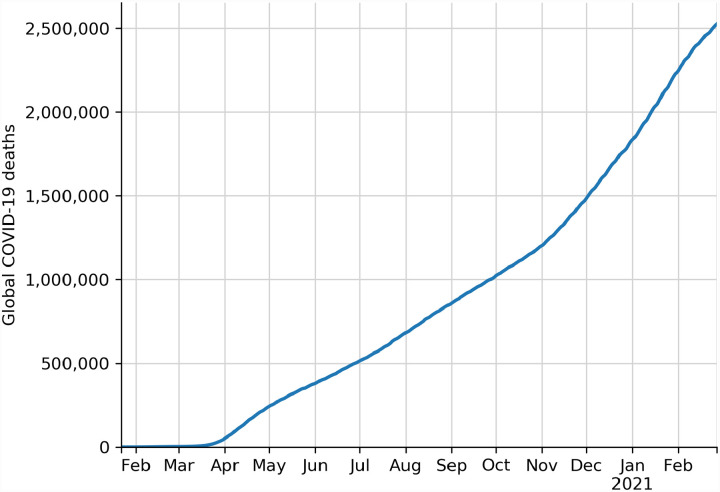
Cumulative global COVID-19 deaths since January 22, 2020. Data are from the COVID-19 Data Repository by the Center for Systems Science and Engineering at Johns Hopkins University [5].

The novel coronavirus *Severe acute respiratory syndrome-related coronavirus 2* (SARS-CoV-2) emerged in late 2019 and quickly precipitated the worldwide spread of novel coronavirus disease 2019 (COVID-19). COVID-19 is associated with symptoms ranging from none (asymptomatic) to mild to severe, with approximately 2% of patients dying from COVID-19-related complications, such as acute respiratory disease syndrome (ARDS) [1]. The virus is likely spread between people primarily by droplets, with the role of contact and aerosol transmission still in question [2,3]. As a result, public health guidelines have been critical to efforts to control the spread of the virus. However, as of early 2021, COVID-19 remains a significant worldwide concern (Figure 1), with cases in some places surging far above the numbers reported during the initial outbreak in early 2020. Due to the continued threat of the virus and the severity of the disease, the identification and development of prophylactic and therapeutic interventions have emerged as significant international priorities. Both approaches hold valuable potential for controlling the impact of the disease. Prophylactics bolster immunity to prevent an individual from contracting a disease, whereas therapeutics treat a disease in individuals who have already been infected. While a number of vaccines have recently been developed, approved, or are currently being evaluated by the US Food and Drug Administration and other regional and international bodies, vaccination programs only started being rolled out at the very end of 2020 and beginning of 2021, meaning that treatments that palliate symptoms and prevent the most severe outcomes have been necessary for much of 2020 and will continue to be necessary for the foreseeable future [4]. Fortunately, prior developments during other recent pandemics, especially those caused by human coronaviruses (HCoV), have provided a number of hypotheses guiding a biomedical approach to the novel coronavirus infection.

2,525,401 COVID-19 deaths had been reported worldwide as of February 27, 2021 (Figure 1).

### Lessons from Prior HCoV Outbreaks

SARS-CoV-2’s rapid shift from an unknown virus to a significant worldwide threat closely parallels the emergence of *Severe acute respiratory syndrome-related coronavirus* (SARS-CoV-1). The first documented case of COVID-19 was reported in Wuhan, China in November 2019, and the disease quickly spread worldwide during the early months of 2020. Similarly, the first case of SARS was reported in November 2002 in the Guangdong Province of China, and it spread within China and then into several countries across continents over the following months [6,7]. In fact, genome sequencing quickly revealed the virus causing COVID-19 to be a novel betacoronavirus closely related to SARS-CoV-1 [8].

There are many similarities but also some differences in the characteristics of the two viruses that determine how they spread. SARS-CoV-1 infection is severe, with an estimated death rate for SARS of 9.5% [6], while estimates of the death rate associated with COVID-19 are much lower, at approximately 2% [1]. SARS-CoV-1 is highly contagious via droplet transmission and has a basic reproduction number (R_0_) of 4 (i.e., each person infected was estimated to infect four other people) [6]. SARS-CoV-2 also appears to be spread primarily by droplet transmission [2,3], and most estimates of its R_0_ fall between 2.5 and 3 [1]. However, the 17-year difference in the timing of these two outbreaks has led to some major differences in the tools available for the international community’s response. At the time that SARS-CoV-1 emerged, no new HCoV had been identified in almost 40 years [7]. The identity of the virus underlying the SARS disease remained unknown until April of 2003, when the SARS-CoV-1 virus was characterized through a worldwide scientific effort spearheaded by the World Health Organization (WHO) [7]. In contrast, the SARS-CoV-2 genomic sequence was released on January 3, 2020 [8], only days after the international community became aware of the novel pneumonia-like illness now known as COVID-19. While SARS-CoV-1 belonged to a distinct lineage from the two other HCoVs known at the time of its discovery [6], SARS-CoV-2 is closely related to SARS-CoV-1 and a more distant relative of another HCoV characterized in 2012, *Middle East respiratory syndrome-related coronavirus* [9,10].

Despite their phylogenetic similarity, SARS-CoV-2 emerged under very different circumstances than SARS-CoV-1 in terms of scientific knowledge about HCoVs. The trajectories of the pandemics associated with each of the viruses have also diverged significantly. By July 2003, the SARS outbreak was officially determined to be under control, with the success credited to infection management practices such as mask wearing [7]. In contrast, Middle East respiratory syndrome (MERS) is still circulating and remains a concern; although the fatality rate is very high at almost 35%, the disease is much less easily transmitted, as its R_0_ has been estimated to be 1 [6]. The low R_0_ in combination with public health practices allowed for its spread to be contained [6]. Neither of these trajectories are comparable to that of SARS-CoV-2, which remains a serious threat worldwide more than a year after the first cases of COVID-19 emerged.

Current results suggest that pharmaceutical interventions for COVID-19 appear to be more successful than the previous efforts to develop therapeutics for SARS and MERS. Care for SARS and MERS patients prioritized supportive care and symptom management [6]. To the extent that clinical treatments for SARS and MERS were explored, there is generally a lack of evidence supporting their efficacy. For example, Ribavirin is an antiviral that was often used in combination with corticosteroids and sometimes interferon (IFN) medications to treat SARS and MERS [7], but its effects have been found to be inconclusive in retrospective and *in vitro* analyses of SARS and the SARS-CoV-1 virus, respectively [7]. IFNs and Ribavirin have shown promise in *in vitro* analyses of MERS, but their clinical effectiveness remains unknown [7]. Therefore, only limited pharmaceutical advances from prior HCoV outbreaks can be adopted to COVID-19. Importantly, though, prior analyses of the virological and pathogenic properties of SARS-CoV-1 and *Middle East respiratory syndrome-related coronavirus* (MERS-CoV) have provided a strong foundation for the development of hypotheses about SARS-CoV-2 that have served to accelerated the development and identification of potential therapeutic approaches. Even during the early months of the pandemic, a number of studies have emerged investigating the potential effects of drugs with mechanisms suggested based on prior understanding of coronavirus-host interactions. Initially, these were primarily observational studies, which simply compare groups of patients who did and did not receive a treatment to determine whether it may have an effect. However, these studies are subject to confounding, and randomized controlled trials are the standard means of assessing the effects of medications. In randomized controlled trials, patients are prospectively and randomly assigned to treatment conditions, allowing for much stronger interpretations to be drawn; however, data from these trials takes much longer to collect. Both have proven to be important sources of information during the COVID-19 crisis, but as more results are released from randomized controlled trials, more definitive answers are becoming available about various proposed therapeutics.

### Overview of Therapeutic Approaches

Therapeutic approaches to the current pandemic can utilize two potential avenues: they can reduce the symptoms that are harmful to COVID-19 patients, or they can directly target the virus to hinder the spread of infection. The goal of the former is to reduce the severity and risks of an active infection, while for the latter, it is to inhibit the replication of the virus once an individual is infected. A variety of symptom profiles with a range of severity are associated with COVID-19, many of which are not life-threatening. A study of COVID-19 patients in a hospital in Berlin, Germany found that the symptoms associated with the highest risk of death included infection-related symptoms, such as sepsis, respiratory symptoms such as ARDS, and cardiovascular failure or pulmonary embolism [11]. Therapeutics that reduce the risks associated with these severe outcomes hold particular potential to reduce the pandemic death toll, while therapeutics that directly target the virus itself would hold the potential to prevent people infected with SARS-CoV-2 from developing potentially damaging symptoms. The treatments in this latter category typically fall into the broad category of antivirals. Antiviral therapies hinder the spread of a virus within the host, rather than destroying existing copies of the virus, and these drugs can vary in their specificity to a narrow or broad range of viral targets. For both categories, uncertainty often surrounds the treatments’ exact mechanisms of action, as most therapies have secondary or off-target effects.

**Figure 2:**
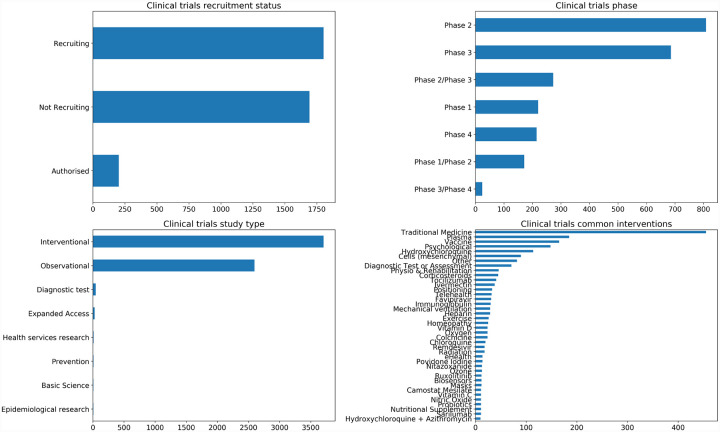
COVID-19 clinical trials. There are 6,417 COVID-19 clinical trials of which 3,706 are interventional. The study types include only types used in at least five trials. Interventional trials only are analyzed in the figures depicting status, phase, and intervention. Of the interventional trials, 98 trials had reported results as of November 9, 2020. Recruitment status and trial phase are shown only for interventional trials in which the status or phase is recorded. The common interventions are all interventions used in at least ten trials. Combinations of interventions, such as Hydroxychloroquine + Azithromycin, are tallied separately from the individual interventions. Trials data are from the University of Oxford Evidence-Based Medicine Data Lab’s COVID-19 TrialsTracker [12].

A large number of clinical trials investigating a range of possible therapeutics for COVID-19 are currently in progress or have already been completed (Figure 2). The purpose of this review is to critically appraise the literature surrounding a subset of clinical trials and to evaluate a range of approaches to repurpose existing or develop novel approaches to the mitigation and treatment of coronavirus infections. The treatments evaluated here are classified according to their biological properties, specifically whether they are biologics (produced from components of organisms) or small molecules. Small molecule drugs include drugs targeted at viral particles, drugs targeted at host proteins, and broad spectrum pharmaceuticals, while biologics include antibodies and interferons. As we cover vaccines in a separate manuscript [4], we only consider therapies for the prevention of SARS-CoV-2 infection or COVID-19 in a very limited context here, for example when a drug is studied to see whether it can prevent infection or disease in recently-exposed individuals. As results become available from additional clinical trials, we will continue to update this manuscript to keep pace with the current understanding of which therapeutics may be effective against SARS-CoV-2 or COVID-19.

## Small Molecule Drugs

Small molecules are synthesized compounds of low molecular weight, typically less than 1 kilodalton (kDa) [13]. Small-molecule pharmaceutical agents have been a backbone of drug development since the discovery of penicillin in the early twentieth century [14]. It and other antibiotics have long been among the best known applications of small molecules to therapeutics, but biotechnological developments such as the prediction of protein-protein interactions have facilitated advances in precise targeting of specific structures using small molecules [14]. Small molecule drugs today encompass a wide range of therapeutics beyond antibiotics, including antivirals, protein inhibitors, and many broad-spectrum pharmaceuticals.

### Small Molecule Antivirals

**Figure 3:**
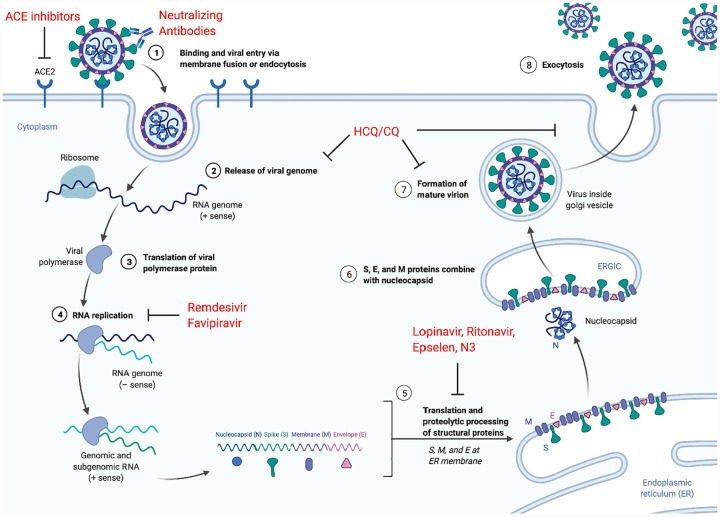
Mechanism of Action for Potential Therapeutics Potential therapeutics currently being studied can target the SARS-CoV-2 virus or modify the host environment through many different mechanisms. Here, the relationship between the virus and several therapeutics described above are visualized.

Antiviral drugs against SARS-CoV-2 are designed to inhibit replication of a virus within an epithelial host cell. This process requires inhibiting the replication cycle of a virus by disrupting one of six fundamental steps [15] (Figure 3). In the first of these steps, the virus attaches to and enters the host cell through endocytosis. Then the virus undergoes uncoating, which is classically defined as the release of viral contents into the host cell. Next, the viral genetic material enters the nucleus where it gets replicated during the biosynthesis stage. During the assembly stage, viral proteins are translated, allowing new viral particles to be assembled. In the final step new viruses are released into the extracellular environment. Many antiviral drugs are designed to inhibit the replication of viral genetic material during the biosynthesis step. Unlike DNA viruses, which can use the host enzymes to propagate themselves, RNA viruses like SARS-CoV-2 depend on their own polymerase, the RNA-dependent RNA polymerase (RdRP), for replication [16,17]. Targeting the RdRP is therefore an effective strategy for antivirals against RNA viruses and is the proposed mechanism underlying the treatment of SARS and MERS with Ribavirin [18]. However, although antivirals are designed to target a virus, they can also impact other processes in the host and may have unintended effects. Therefore, these therapeutics must be evaluated for both efficacy and safety.

#### Nucleoside and Nucleotide Analogs

##### Favipiravir

Favipiravir (Avigan), also known as T-705, was discovered by Toyama Chemical Co., Ltd. [19]. The drug was found to be effective at blocking viral amplification in several influenza subtypes as well as other RNA viruses, such as *Flaviviridae* and *Picornaviridae*, through a reduction in plaque formation [20] and viral replication in Madin-Darby canine kidney cells [21]. Furthermore, inoculation of mice with favipiravir was shown to increase survival of influenza infections [20,21]. In 2014, the drug was approved in Japan for the treatment of influenza that was resistant to conventional treatments like neuraminidase inhibitors [22]. Favipiravir (6-fluoro-3-hydroxy-2-pyrazinecarboxamide) acts as a purine and purine nucleoside analogue that inhibits viral RNA polymerase in a dose-dependent manner across a range of RNA viruses, including influenza viruses [23,24,25,26,27]. Nucleotides and nucleosides are the natural building blocks for RNA synthesis. Because of this, modifications to nucleotides and nucleosides can disrupt key processes including replication [28]. Biochemical experiments showed that favipiravir was recognized as a purine nucleoside analogue and incorporated into the viral RNA template. A single incorporation does not influence RNA transcription; however, multiple events of incorporation lead to the arrest of RNA synthesis [29]. Evidence for T-705 inhibiting viral RNA polymerase are based on time-of-drug addition studies that found that viral loads were reduced with the addition of favipiravir in early times post-infection [23,26,27].

The effectiveness of favipiravir for treating patients with COVID-19 is currently under investigation. An open-label, nonrandomized, before-after controlled study was recently conducted [30]. The study included 80 COVID-19 patients (35 treated with favipiravir, 45 control) from the isolation ward of the National Clinical Research Center for Infectious Diseases (The Third People’s Hospital of Shenzhen), Shenzhen, China. The patients in the control group were treated with other antivirals, such as lopinavir and ritonavir. It should be noted that although the control patients received antivirals, two subsequent large-scale analyses, the WHO Solidarity trial and the Randomized Evaluation of COVID-19 Therapy (RECOVERY) trial, identified no effect of lopinavir or of a lopinavir-ritonavir combination, respectively, on the metrics of COVID-19-related mortality that each assessed [31,32,33]. Treatment was applied on days 2–14; treatment stopped either when viral clearance was confirmed or at day 14. The efficacy of the treatment was measured by, first, the time until viral clearance using Kaplan-Meier survival curves, and, second, the improvement rate of chest computed tomography (CT) scans on day 14 after treatment. The study found that favipiravir increased the speed of recovery, measured as viral clearance from the patient by RT-PCR, with patients receiving favipiravir recovering in four days compared to 11 days for patients receiving antivirals such as lopinavir and ritonavir. Additionally, the lung CT scans of patients treated with favipiravir showed significantly higher improvement rates (91%) on day 14 compared to control patients (62%, *p* = 0.004). However, there were adverse side effects in 4 (11%) favipiravir-treated patients and 25 (56%) control patients. The adverse side effects included diarrhea, vomiting, nausea, rash, and liver and kidney injury. Despite the study reporting clinical improvement in favipiravir-treated patients, several study design issues are problematic and lower confidence in the overall conclusions. For example, the study was neither randomized nor blinded. Moreover, the selection of patients did not take into consideration important factors such as previous clinical conditions or sex, and there was no age categorization. Additionally, it should be noted that this study was temporarily retracted and then restored without an explanation [34].

In late 2020 and early 2021, the first randomized controlled trials of favipiravir for the treatment of COVID-19 released results [35,36,37]. The first [35] used a randomized, controlled, open-label design to compare two drugs, favipiravir and baloxavir marboxil, to standard of care (SOC) alone. Here, SOC included antivirals such as lopinavir/ritonavir and was administered to all patients. The primary endpoint analyzed was viral clearance at day 14. The sample size for this study was very small, with 29 total patients enrolled, and no significant effect of the treatments was found for the primary or any of the secondary outcomes analyzed, which included mortality. The second study [36] was larger, with 96 patients enrolled, and included only individuals with mild to moderate symptoms who were randomized into two groups: one receiving chloroquine (CQ) in addition to SOC, and the other receiving favipiravir in addition to SOC. This study reported a non-significant trend for patients receiving favipiravir to have a shorter hospital stay (13.29 days compared to 15.89 for CQ, *p* = 0.06) and less likelihood of progressing to mechanical ventilation (*p* = 0.118) or to an oxygen saturation < 90% (*p* = 0.129). These results, combined with the fact that favipiravir was being compared to CQ, which is now widely understood to be ineffective for treating COVID-19, thus do not suggest that favipiravir was likely to have had a strong effect on these outcomes. On the other hand, another trial of 60 patients reported a significant effect of favipiravir on viral clearance at four days (a secondary endpoint), but not at 10 days (the primary endpoint) [37]. This study, as well as a prior study of favipiravir [38], also reported that the drug was generally well-tolerated. Thus, in combination, these small studies suggest that the effects of favipiravir as a treatment for COVID-19 cannot be determined based on the available evidence, but additionally, none raise major concerns about the safety profile of the drug.

##### Remdesivir

Remdesivir (GS-5734) is an intravenous antiviral that was proposed by Gilead Sciences as a possible treatment for Ebola virus disease (EVD). At the outset of the COVID-19 pandemic, it did not have any have any FDA-approved use. However, on May 1, 2020, the FDA issued an Emergency Use Authorization (EUA) for remdesivir for the treatment of hospitalized COVID-19 patients [39]. The EUA was based on information from two clinical trials, NCT04280705 and NCT04292899 [40,41,42,43]. Remdesivir is metabolized to GS-441524, an adenosine analog that inhibits a broad range of polymerases and then evades exonuclease repair, causing chain termination [44,45,46]. A clinical trial in the Democratic Republic of Congo found some evidence of effectiveness against EVD, but two antibody preparations were found to be more effective, and remdesivir was not pursued [47]. Remdesivir also inhibits polymerase and replication of the coronaviruses MERS-CoV and SARS-CoV-1 in cell culture assays with submicromolar IC50s [48]. It has also been found to inhibit SARS-CoV-2, showing synergy with CQ *in vitro* [46].

Remdesivir was first used on some COVID-19 patients under compassionate use guidelines [49,50]. All were in late stages of COVID-19 infection, and initial reports were inconclusive about the drug’s efficacy. Gilead Sciences, the maker of remdesivir, led a recent publication that reported outcomes for compassionate use of the drug in 61 patients hospitalized with confirmed COVID-19. Here, 200 mg of remdesivir was administered intravenously on day 1, followed by a further 100 mg/day for 9 days [43]. There were significant issues with the study design, or lack thereof. There was no randomized control group. The inclusion criteria were variable: some patients only required low doses of oxygen, while others required ventilation. The study included many sites, potentially with variable inclusion criteria and treatment protocols. The patients analyzed had mixed demographics. There was a short follow-up period of investigation. Eight patients were excluded from the analysis mainly due to missing post-baseline information; thus, their health was unaccounted for. Therefore, even though the study reported clinical improvement in 68% of the 53 patients ultimately evaluated, due to the significant issues with study design, it could not be determined whether treatment with remdesivir had an effect or whether these patients would have recovered regardless of treatment. Another study comparing 5- and 10-day treatment regimens reported similar results but was also limited because of the lack of a placebo control [52]. These studies did not alter the understanding of the efficacy of remdesivir in treating COVID-19, but the encouraging results provided motivation for placebo-controlled studies.

Remdesivir was later tested in a double-blind placebo-controlled phase 3 clinical trial performed at 60 trial sites, 45 of which were in the United States [41,42]. The trial recruited 1,062 patients and randomly assigned them to placebo treatment or treatment with remdesivir. Patients were stratified for randomization based on site and the severity of disease presentation at baseline [42]. The treatment was 200 mg on day 1, followed by 100 mg on days 2 through 10. Data was analyzed from a total of 1,059 patients who completed the 29-day course of the trial, with 517 assigned to remdesivir and 508 to placebo [42]. The two groups were well matched demographically and clinically at baseline. Those who received remdesivir had a median recovery time of 10 days, as compared with 15 days in those who received placebo (rate ratio for recovery, 1.29; 95% confidence interval (CI), 1.12 to 1.49; *p* < 0.001). The Kaplan-Meier estimates of mortality by 14 days were 6.7% with remdesivir and 11.9% with placebo, with a hazard ratio (HR) for death of 0.55 and a 95% CI of 0.36 to 0.83, and at day 29, remdesivir corresponded to 11.4% and the placebo to 15.2% (HR: 0.73; 95% CI, 0.52 to 1.03). Serious adverse events were reported in 131 of the 532 patients who received remdesivir (24.6%) and in 163 of the 516 patients in the placebo group (31.6%). This study also reported an association between remdesivir administration and both clinical improvement and a lack of progression to more invasive respiratory intervention in patients receiving non-invasive and invasive ventilation at randomization [42]. Largely on the results of this trial, the FDA reissued and expanded the EUA for remdesivir for the treatment of hospitalized COVID-19 patients ages twelve and older [39]. Additional clinical trials [46,53,54,55,56] are currently underway to evaluate the use of remdesivir to treat COVID-19 patients at both early and late stages of infection and in combination with other drugs (Figure 2). As of October 22, 2020, remdesivir received FDA approval based on three clinical trials [57].

However, results suggesting no effect of remdesivir on survival were reported by the WHO Solidarity trial [31]. This large-scale, open-label trial enrolled 11,330 adult in-patients at 405 hospitals in 30 countries around the world [31]. Patients were randomized in equal proportions into four experimental conditions and a control condition, corresponding to four candidate treatments for COVID-19 and SOC, respectively; no placebo was administered. The 2,750 patients in the remdesivir group were administered 200 mg intravenously on the first day and 100 mg on each subsequent day until day 10 and assessed for in-hospital death (primary endpoint), duration of hospitalization, and progression to mechanical ventilation. There were also 2,708 control patients who would have been eligible and able to receive remdesivir were they not assigned to the control group. A total of 604 patients among these two cohorts died during initial hospitalization, with 301 in the remdesivir group and 303 in the control group. The rate ratio of death between these two groups was therefore not significant (0.95, *p* = 0.50), suggesting that the administration of remdesivir did not affect survival. The two secondary analyses similarly did not find any effect of remdesivir. Additionally, the authors compared data from their study with data from three other studies of remdesivir (including [42]) stratified by supplemental oxygen status. A meta-analysis of the four studies yielded an overall rate ratio for death of 0.91 (*p* = 0.20). These results thus do not support the previous findings that remdesivir reduced median recovery time and mortality risk in COVID-19 patients.

In response to the results of the Solidarity trial, Gilead, which manufactures remdesivir, released a statement pointing to the fact that the Solidarity trial was not placebo-controlled or double-blind and at the time of release, the statement had not been peer reviewed [58]; these sentiments have been echoed elsewhere [59]. Other critiques of this study have noted that antivirals are not typically targeted at patients with severe illness, and therefore remdesivir could be more beneficial for patients with mild rather than severe cases [33,60]. However, the publication associated with the trial sponsored by Gilead did purport an effect of remdesivir on patients with severe disease, identifying an 11 versus 18 day recovery period (rate ratio for recovery: 1.31, 95% CI 1.12 to 1.52) [42]. Additionally, a smaller analysis of 598 patients, of whom two-thirds were randomized to receive remdesivir for either 5 or 10 days, reported a small effect of treatment with remdesivir for five days relative to standard of care in patients with moderate COVID-19 [61]. These results suggest that remdesivir could improve outcomes for patients with moderate COVID-19, but that additional information would be needed to understand the effects of different durations of treatment. Therefore, the Solidarity trial may point to limitations in the generalizability of other research on remdesivir, especially since the broad international nature of the Solidarity clinical trial, which included countries with a wide range of economic profiles and a variety of healthcare systems, provides a much-needed global perspective in a pandemic [33]. On the other hand, only 62% of patients in the Solidarity trial were randomized on the day of admission or one day afterwards [31], and concerns have been raised that differences in disease progression could influence the effectiveness of remdesivir [33]. Despite the findings of the Solidarity trial, remdesivir remains available for the treatment of COVID-19 in many places. Remdesivir has also been investigated in combination with other drugs, such as baricitinib, which is an inhibitor of Janus kinase 1 and 2 [62]; the FDA has issued an EUA for the combination of remdesivir and baricitinib in adult and pediatric patients [63]. Follow-up studies are needed and, in many cases, are underway to further investigate remdesivir-related outcomes.

Similarly, the extent to which the remdesivir dosing regimen could influence outcomes continues to be under consideration. A randomized, open-label trial compared the effect of remdesivir on 397 patients with severe COVID-19 over 5 versus 10 days [40,52], complementing the study that found that a 5-day course of remdesivir improved outcomes for patients with moderate COVID-19 but a 10-day course did not [61]. Patients in the two groups were administered 200 mg of remdesivir intravenously on the first day, followed by 100 mg on the subsequent four or nine days, respectively. The two groups differed significantly in their clinical status, with patients assigned to the 10-day group having more severe illness. This study also differed from most because it included not only adults, but also pediatric patients as young as 12 years old. It reported no significant differences across several outcomes for patients receiving a 5-day or 10-day course, when correcting for baseline clinical status. The data did suggest that the 10-day course might reduce mortality in the most severe patients at day 14, but the representation of this group in the study population was too low to justify any conclusions [52]. Thus, additional research is also required to determine whether the dosage and duration of remdesivir administration influences outcomes.

In summary, remdesivir is the first FDA approved anti-viral against SARS-CoV-2 as well as the first FDA approved COVID-19 treatment. Early investigations of this drug established proof of principle that drugs targeting the virus can benefit COVID-19 patients. It also shows proof of principle that SARS-CoV-2 can be targeted at the level of viral replication, since remdesivir targets the viral RNA polymerase at high potency. Moreover, one of the most successful strategies for developing therapeutics for viral diseases is to target the viral replication machinery, which are typically virally encoded polymerases. Small molecule drugs targeting viral polymerases are the backbones of treatments for other viral diseases including human immunodeficiency virus (HIV) and herpes. Notably, the HIV and herpes polymerases are a reverse transcriptase and a DNA polymerase, respectively, whereas SARS-CoV-2 encodes an RdRP, so most of the commonly used polymerase inhibitors are not likely to be active against SARS-CoV-2. In clinical use, polymerase inhibitors show short term benefits for HIV patients, but for long term benefits they must be part of combination regimens. They are typically combined with protease inhibitors, integrase inhibitors, and even other polymerase inhibitors. Additional clinical trials of remdesivir in different patient pools and in combination with other therapies will refine its use in the clinic.

#### Protease Inhibitors

Several studies showed that viral proteases play an important role in the life cycle of viruses, including coronaviruses, by modulating the cleavage of viral polyprotein precursors [64]. Several FDA-approved drugs target proteases, including lopinavir and ritonavir for HIV infection and simeprevir for hepatitis C virus infection. In particular, serine protease inhibitors were suggested for the treatment of SARS and MERS viruses [65]. Recently, a study [66] suggested that camostat mesylate, an FDA-approved protease inhibitor could block the entry of SARS-CoV-2 into lung cells *in vitro*. Thus far, investigation of possible protease inhibitors that could work against SARS-CoV-2 has been driven by computational predictions.

Computer-aided design allowed for the development of a Michael acceptor inhibitor, now known as N3, to target a protease critical to SARS-CoV-2 replication. Discovery of the N3 mechanism arose from interest in the two polyproteins encoded by the SARS-CoV-2 replicase gene, pp1a and pp1ab, that are critical for viral replication and transcription [67]. These polyproteins must undergo proteolytic processing. This processing is usually conducted by Mpro, a 33.8-kDa SARS-CoV-2 protease that is therefore fundamental to viral replication and transcription. N3 was designed computationally [68] to bind in the substrate binding pocket of the Mpro protease of SARS-like coronaviruses [69], therefore inhibiting proteolytic processing. Subsequently, the structure of N3-bound SARS-CoV-2 Mpro was solved [67], confirming the computational prediction. N3 was tested *in vitro* on SARS-CoV-2-infected Vero cells, which belong to a line of cells established from the kidney epithelial cells of an African green monkey, and was found to inhibit SARS-CoV-2 [67].

Although N3 is a strong inhibitor of SARS-CoV-2 *in vitro*, its safety and efficacy still need to be tested in healthy volunteers and patients. After the design and confirmation of N3 as a highly potent Michael acceptor inhibitor and the identification of Mpro’s structure [67,70], 10,000 compounds were screened for their *in vitro* anti-Mpro activity. The six leads that were identified were ebselen, disulfiram, tideglusib, carmofur, and PX-12. *In vitro* analysis revealed that ebselen had the strongest potency in reducing the viral load in SARS-CoV-2-infected Vero cells [67]. Ebselen is an organoselenium compound with anti-inflammatory and antioxidant properties [71]. It has been proposed as a possible treatment for conditions ranging from bipolar disorder to diabetes to heart disease [71], and a preliminary investigation of ebselen as a treatment for noise-induced hearing loss provided promising reports of its safety [72]. For COVID-19, the NSP5 in SARS-CoV-2 contains a cysteine at the active site of Mpro, and ebselen is able to inactivate the protease by bonding covalently with this cysteine to form a selenosulfide [71,73]. Interestingly there has been some argument that selenium deficiency may be associated with more severe COVID-19 outcomes [74,75,76], possibly indicating that its antioxidative properties are protective [73]. On the other hand, ebselen and the other compounds identified are likely to be promiscuous binders, which could diminish their therapeutic potential [67]. While there is clear computational and *in vitro* support for ebselen’s potential as a COVID-19 therapeutic, results from clinical trials are not yet available for this compound. However, as of July 2020, phase II clinical trials commenced to assess the effects of SPI-1005, an investigational drug from Sound Pharmaceuticals that contains ebselen [77], on 60 adults presenting with each of moderate [78] and severe [79] COVID-19.

In summary, N3 is a computationally designed molecule that inhibits the viral transcription through inhibiting Mpro. Ebselen is both a strong Mpro inhibitor and strong inhibitor of viral replication *in vitro* that was found to reduce SARS-CoV-2 viral load even more effectively than N3. Ebselen is a promising compound since its safety has been demonstrated in other indications. However, ebselen may be a false positive, since it is a promiscuous compound that can have many targets [80]. Therefore, the results of ongoing clinical trials are expected to help establish whether compounds with higher specificity are required.

### Broad-Spectrum Pharmaceuticals

When a virus enters a host, the host becomes the virus’s environment. Therefore, the state of the host can also influence the virus’s ability to replicate and spread. Traditionally, viral targets have been favored for pharmaceutical interventions because altering host processes is likely to be less specific than targeting the virus directly [81]. On the other hand, targeting the host offers potential for a complementary strategy to antivirals that could broadly limit the ability of viruses to replicate [81]. As a result, therapeutic approaches that target host proteins have become an area of interest for SARS-CoV-2. Viral entry receptors in particular have been identified as a potential target. Entry of SARS-CoV-2 into the cell depends on binding to angiotensin-converting enzyme 2 (ACE2), which is catalyzed by the enzyme encoded by *TMPRSS2* [66]. In principle, drugs that reduce the expression of these proteins or sterically hinder viral interactions with them might reduce viral entry into cells.

Due to the urgent nature of the COVID-19 pandemic, many of the pharmaceutical agents that have been widely publicized as having possible therapeutic or prophylactic effects are broad-spectrum pharmaceuticals that pre-date the COVID-19 pandemic. These treatments are not specifically targeted at the virus itself or at the host receptors it relies on, but rather induce broad shifts in host biology that are hypothesized to be potential inhibitors of the virus. In most cases, interest in particular candidate medications arises because they are already available for other purposes. However, the fact that the targets of these agents are non-specific means that the mechanism of action can appear to be relevant to COVID-19 without a therapeutic or prophylactic effect being observed in clinical trials. This category of drugs has also received significant attention from the media and general public, often before rigorous testing has been able to determine their effectiveness against SARS-CoV-2.

#### ACE Inhibitors and Angiotensin II Receptor Blockers

Angiotensin-converting enzyme (ACE) inhibitors and angiotensin II receptor blockers (ARBs) are among today’s most commonly prescribed medications, often being used to control blood pressure [82,83]. In the United States, for example, they are prescribed well over 100,000,000 times annually [84]. Data from some animal models suggest that several, but not all, ACE inhibitors (ACEIs) and several ARBs increase ACE2 expression in the cells of some organs [85]. Clinical studies have not established whether plasma ACE2 expression is increased in humans treated with these medications [86]. While randomized clinical trials are ongoing, a variety of observational studies have examined the relationship between exposure to ACEIs or ARBs and outcomes in patients with COVID-19. An observational study of the association of exposure to ACEIs or ARBs with outcomes in COVID-19 was retracted from the *New England Journal of Medicine* [87] due to concerns related to data availability [88]. Clinical trials are needed because the findings of the various observational studies bearing on this topic cannot be interpreted as indicating a protective effect of the drug [89,90]. Several clinical trials testing the effects of ACEIs or ARBs on COVID-19 outcomes are ongoing [91,92,93,94,95,96,97].

Two of these analyses [91,97] have reported no effect of continuing or discontinuing ARBs and ACEIs on patients admitted to the hospital for COVID-19. The first, known as REPLACE COVID [98], was a randomized, open-label study that enrolled patients who were admitted to the hospital for COVID-19 and were taking an ACEI at the time of admission. They enrolled 152 patients at 20 hospitals across seven countries and randomized them into two arms, continuation (n=75) and discontinuation (n=77). The primary outcome evaluated was a global rank score that integrated several dimensions of illness. The components of this global rank score, such as time to death and length of mechanical ventilation, were evaluated as secondary endpoints. This analysis reported no differences between the two groups in the primary or any of the secondary outcomes.

Similarly, a second study [99] used a randomized, open-label design to examine the effects of continuing versus discontinuing ARBs and ACEIs on patients hospitalized for mild to moderate COVID-19 at 29 hospitals in Brazil. This study enrolled 740 patients but had to exclude one trial site from all analyses due to the discovery of violations of Good Clinical Trial practice and data falsification. After this exclusion, 659 patients remained, with 334 randomized to discontinuation and 325 to continuation. In this study, the primary endpoint analyzed was the number of days that patients were alive and not hospitalized within 30 days of enrollment. The secondary outcomes included death (including in-hospital death separately), number of days hospitalized, and specific clinical outcomes such as heart failure or stroke. Once again, no significant differences were found between the two groups. Initial studies of randomized interventions therefore suggest that ACEIs and ARBs are unlikely to affect COVID-19 outcomes. These results are also consistent with findings from observational studies (summarized in [98]). Additional information about ACE2, observational studies of ACEIs and ARBs in COVID-19, and clinical trials on this topic have been summarized [100]. Therefore, despite the promising potential mechanism, initial results have not provided support for ACEIs and ARBs as therapies for COVID-19.

#### Hydroxychloroquine and Chloroquine

CQ and hydroxychloroquine (HCQ) are lysosomotropic agents, meaning they are weak bases that can pass through the plasma membrane. Both drugs increase cellular pH by accumulating in their protonated form inside lysosomes [101,102]. These drugs are used for the treatment and prophylaxis of malaria, as well as the treatment of lupus erythematosus and rheumatoid arthritis in adults [103]. This shift in pH inhibits the breakdown of proteins and peptides by the lysosomes during the process of proteolysis [102]. A number of mechanisms have been proposed through which these drugs could influence the immune response to pathogen challenge. For example, CQ/HCQ can interfere with digestion of antigens within the lysosome and inhibit CD4 T-cell stimulation while promoting the stimulation of CD8 T-cells [102]. CQ/HCQ can also decrease the production of certain key cytokines involved in the immune response, including interleukin-6 (IL-6), and inhibit the stimulation of Toll-like receptors (TLR) and TLR signaling [102]. The drugs also have anti-inflammatory and photoprotective effects and may also affect rates of cell death, blood clotting, glucose tolerance, and cholesterol levels [102].

Interest in CQ and HCQ for treating COVID-19 was catalyzed by a mechanism observed in *in vitro* studies of both SARS-CoV-1 and SARS-CoV-2. In one study, CQ inhibited viral entry of SARS-CoV-1 into Vero E6 cells, a cell line that was derived from Vero cells in 1968, through the elevation of endosomal pH and the terminal glycosylation of ACE2 [104]. Increased pH within the cell, as discussed above, inhibits proteolysis, and terminal glycosylation of ACE2 is thought to interfere with virus-receptor binding. An *in vitro* study of SARS-CoV-2 infection of Vero cells found both HCQ and CQ to be effective in inhibiting viral replication, with HCQ being more potent [105]. Additionally, an early case study of three COVID-19 patients reported the presence of antiphospholipid antibodies in all three patients [106]. Antiphospholipid antibodies are central to the diagnosis of the antiphospholipid syndrome, a disorder that HCQ has often been used to treat [107,108,109]. Because the 90% effective concentration (EC_90_) of CQ in Vero E6 cells (6.90 μM) can be achieved in and tolerated by rheumatoid arthritis (RA) patients, it was hypothesized that it might also be possible to achieve the effective concentration in COVID-19 patients [110]. Additionally, clinical trials have reported HCQ to be effective in treating HIV [111] and chronic Hepatitis C [112]. Together, these studies triggered initial enthusiasm about the therapeutic potential for HCQ and CQ against COVID-19. HCQ/CQ has been proposed both as a treatment for COVID-19 and a prophylaxis against SARS-CoV-2 exposure, and trials often investigated these drugs in combination with azithromycin (AZ) and/or zinc supplementation. However, as more evidence has emerged, it has become clear that HCQ/CQ offer no benefits against SARS-CoV-2 or COVID-19.

#### Trials Assessing Therapeutic Administration of HCQ/CQ

The initial study evaluating HCQ as a treatment for COVID-19 patients was published on March 20, 2020 by Gautret et al. [113]. This non-randomized, non-blinded, non-placebo clinical trial compared HCQ to SOC in 42 hospitalized patients in southern France. It reported that patients who received HCQ showed higher rates of virological clearance by nasopharyngeal swab on days 3–6 when compared to SOC. This study also treated six patients with both HCQ + AZ and found this combination therapy to be more effective than HCQ alone. However, the design and analyses used showed weaknesses that severely limit interpretability of results, including the small sample size and the lack of: randomization, blinding, placebo (no “placebo pill” given to SOC group), Intention-To-Treat analysis, correction for sequential multiple comparisons, and trial pre-registration. Furthermore, the trial arms were entirely confounded by hospital and there were false negative outcome measurements (see [114]). Two of these weaknesses are due to inappropriate data analysis and can therefore be corrected *post hoc* by recalculating the p-values (lack of Intention-To-Treat analysis and multiple comparisons). However, all other weaknesses are fundamental design flaws and cannot be corrected for. Thus, the conclusions cannot be generalized outside of the study. The International Society of Antimicrobial Chemotherapy, the scientific organization that publishes the journal where the article appeared, subsequently announced that the article did not meet its expected standard for publications [115], although it has not been officially retracted.

Because of the preliminary data presented in this study, HCQ treatment was subsequently explored by other researchers. About one week later, a follow-up case study reported that 11 consecutive patients were treated with HCQ + AZ using the same dosing regimen [116]. One patient died, two were transferred to the intensive care unit (ICU), and one developed a prolonged QT interval, leading to discontinuation of HCQ + AZ administration. As in the Gautret et al. study, the outcome assessed was virological clearance at day 6 post-treatment, as measured from nasopharyngeal swabs. Of the ten living patients on day 6, eight remained positive for SARS-CoV-2 RNA. Like in the original study, interpretability was severely limited by the lack of a comparison group and the small sample size. However, these results stand in contrast to the claims by Gautret et al. that all six patients treated with HCQ + AZ tested negative for SARS-CoV-2 RNA by day 6 post-treatment. This case study illustrated the need for further investigation using robust study design to evaluate the efficacy of HCQ and/or CQ.

On April 10, 2020, a randomized, non-placebo trial of 62 COVID-19 patients at the Renmin Hospital of Wuhan University was released [117]. This study investigated whether HCQ decreased time to fever break or time to cough relief when compared to SOC [117]. This trial found HCQ decreased both average time to fever break and average time to cough relief, defined as mild or no cough. While this study improved on some of the methodological flaws in Gautret et al. by randomizing patients, it also had several flaws in trial design and data analysis that prevent generalization of the results. These weaknesses include the lack of placebo, lack of correction for multiple primary outcomes, inappropriate choice of outcomes, lack of sufficient detail to understand analysis, drastic disparities between pre-registration [118] and published protocol (including differences in the inclusion and exclusion criteria, the number of experimental groups, the number of patients enrolled, and the outcome analyzed), and small sample size. The choice of outcomes may be inappropriate as both fevers and cough may break periodically without resolution of illness. Additionally, for these outcomes, the authors reported that 23 of 62 patients did not have a fever and 25 of 62 patients did not have a cough at the start of the study, but the authors failed to describe how these patients were included in a study assessing time to fever break and time to cough relief. It is important to note here that the authors claimed “neither the research performers nor the patients were aware of the treatment assignments.” This blinding seems impossible in a non-placebo trial because at the very least, providers would know whether they were administering a medication or not, and this knowledge could lead to systematic differences in the administration of care. Correction for multiple primary outcomes can be adjusted *post hoc* by recalculating p-values, but all of the other issues were design and statistical weaknesses that cannot be corrected for. Additionally, the observation of drastic disparities between pre-registration and published protocol could indicate p-hacking [119]. The design limitations mean that the conclusions cannot be generalized outside of the study.

A second randomized trial, conducted by the Shanghai Public Health Clinical Center, analyzed whether HCQ increased rates of virological clearance at day 7 in respiratory pharyngeal swabs compared to SOC [120]. This trial was published in Chinese along with an abstract in English, and only the English abstract was read and interpreted for this review. The trial found comparable outcomes in virological clearance rate, time to virological clearance, and time to body temperature normalization between the treatment and control groups. The small sample size is one weakness, with only 30 patients enrolled and 15 in each arm. This problem suggests the study is underpowered to detect potentially useful differences and precludes interpretation of results. Additionally, because only the abstract could be read, other design and analysis issues could be present. Thus, though these studies added randomization to their assessment of HCQ, their conclusions should be interpreted very cautiously. These two studies assessed different outcomes and reached differing conclusions about the efficacy of HCQ for treating COVID-19; the designs of both studies, especially with respect to sample size, meant that no general conclusions can be made about the efficacy of the drug.

Several widely reported studies on HCQ also have issues with data integrity and/or provenance. A Letter to the Editor published in *BioScience Trends* on March 16, 2020 claimed that numerous clinical trials have shown that HCQ is superior to control treatment in inhibiting the exacerbation of COVID-19 pneumonia [121]. This letter has been cited by numerous primary literature, review articles, and media alike [122,123]. However, the letter referred to 15 pre-registration identifiers from the Chinese Clinical Trial Registry. When these identifiers are followed back to the registry, most trials claim they are not yet recruiting patients or are currently recruiting patients. For all of these 15 identifiers, no data uploads or links to publications could be located on the pre-registrations. At the very least, the lack of availability of the primary data means the claim that HCQ is efficacious against COVID-19 pneumonia cannot be verified. Similarly, a recent multinational registry analysis [124] analyzed the efficacy of CQ and HCQ with and without a macrolide, which is a class of antibiotics that includes Azithromycin, for the treatment of COVID-19. The study observed 96,032 patients split into a control and four treatment conditions (CQ with and without a macrolide; HCQ with and without a macrolide). They concluded that treatment with CQ or HCQ was associated with increased risk of *de novo* ventricular arrhythmia during hospitalization. However, this study has since been retracted by *The Lancet* due to an inability to validate the data used [125]. These studies demonstrate that increased skepticism in evaluation of the HCQ/CQ and COVID-19 literature may be warranted, possibly because of the significant attention HCQ and CQ have received as possible treatments for COVID-19 and the politicization of these drugs.

Despite the fact that the study suggesting that CQ/HCQ increased risk of ventricular arrhythmia in COVID-19 patients has now been retracted, previous studies have identified risks associated with HCQ/CQ. A patient with systemic lupus erythematosus developed a prolonged QT interval that was likely exacerbated by use of HCQ in combination with renal failure [126]. A prolonged QT interval is associated with ventricular arrhythmia [127]. Furthermore, a separate study [128] investigated the safety associated with the use of HCQ with and without macrolides between 2000 and 2020. The study involved 900,000 cases treated with HCQ and 300,000 cases treated with HCQ + AZ. The results indicated that short-term use of HCQ was not associated with additional risk, but that HCQ + AZ was associated with an enhanced risk of cardiovascular complications (such as a 15% increased risk of chest pain, calibrated HR = 1.15, 95% CI, 1.05 to 1.26) and a two-fold increased 30-day risk of cardiovascular mortality (calibrated HR = 2.19; 95% CI, 1.22 to 3.94). Therefore, whether studies utilize HCQ alone or HCQ in combination with a macrolide may be an important consideration in assessing risk. As results from initial investigations of these drug combinations have emerged, concerns about the efficacy and risks of treating COVID-19 with HCQ and CQ have led to the removal of CQ/HCQ from SOC practices in several countries [129,130]. As of May 25, 2020, WHO had suspended administration of HCQ as part of the worldwide Solidarity Trial [131], and later the final results of this large-scale trial that compared 947 patients administered HCQ to 906 controls revealed no effect on the primary outcome, mortality during hospitalization (rate ratio: 1.19; *p* = 0.23)

Additional research has emerged largely identifying HCQ/CQ to be ineffective against COVID-19 while simultaneously revealing a number of significant side effects. A randomized, open-label, non-placebo trial of 150 COVID-19 patients was conducted in parallel at 16 government-designated COVID-19 centers in China to assess the safety and efficacy of HCQ [132]. The trial compared treatment with HCQ in conjunction with SOC to SOC alone in 150 infected patients who were assigned randomly to the two groups (75 per group). The primary endpoint of the study was the negative conversion rate of SARS-CoV-2 in 28 days, and the investigators found no difference in this parameter between the groups (estimated difference between SOC plus HCQ and SOC 4.1%; 95% CI, −10.3% to 18.5%). The secondary endpoints were an amelioration of the symptoms of the disease such as axillary temperature ≤36.6°C, SpO2 >94% on room air, and disappearance of symptoms like shortness of breath, cough, and sore throat. The median time to symptom alleviation was similar across different conditions (19 days in HCQ + SOC versus 21 days in SOC, *p* = 0.97). Additionally, 30% of the patients receiving SOC+HCQ reported adverse outcomes compared to 8.8% of patients receiving only SOC, with the most common adverse outcome in the SOC+HCQ group being diarrhea (10% versus 0% in the SOC group, *p* = 0.004). However, there are several factors that limit the interpretability of this study. Most of the enrolled patients had mild-to-moderate symptoms (98%), and the average age was 46. SOC in this study included the use of antivirals (Lopinavir-Ritonavir, Arbidol, Oseltamivir, Virazole, Entecavir, Ganciclovir, and Interferon alfa), which the authors note could influence the results. Thus, they note that an ideal SOC would need to exclude the use of antivirals, but that ceasing antiviral treatment raised ethical concerns at the time that the study was conducted. In this trial, the samples used to test for the presence of the SARS-CoV-2 virus were collected from the upper respiratory tract, and the authors indicated that the use of upper respiratory samples may have introduced false negatives (e.g., [133]). Another limitation of the study that the authors acknowledge was that the HCQ treatment began, on average, at a 16-day delay from the symptom onset. The fact that this study was open-label and lacked a placebo limits interpretation, and additional analysis is required to determine whether HCQ reduces inflammatory response. Therefore, despite some potential areas of investigation identified in *post hoc* analysis, this study cannot be interpreted as providing support for HCQ as a therapeutic against COVID-19. This study provided no support for HCQ against COVID-19, as there was no difference between the two groups in either negative seroconversion at 28 days or symptom alleviation, and in fact, more severe adverse outcomes were reported in the group receiving HCQ.

Additional evidence comes from a retrospective analysis [134] that examined data from 368 COVID-19 patients across all United States Veteran Health Administration medical centers. The study retrospectively investigated the effect of the administration of HCQ (n=97), HCQ + AZ (n=113), and no HCQ (n=158) on 368 patients. The primary outcomes assessed were death and the need for mechanical ventilation. Standard supportive care was rendered to all patients. Due to the low representation of women (N=17) in the available data, the analysis included only men, and the median age was 65 years. The rate of death was 27.8% in the HCQ-only treatment group, 22.1% in the HCQ + AZ treatment group, and 14.1% in the no-HCQ group. These data indicated a statistically significant elevation in the risk of death for the HCQ-only group compared to the no-HCQ group (adjusted HR: 2.61, *p* = 0.03), but not for the HCQ + AZ group compared to the no-HCQ group (adjusted HR: 1.14; *p* = 0.72). Further, the risk of ventilation was similar across all three groups (adjusted HR: 1.43, *p* = 0.48 (HCQ) and 0.43, *p* = 0.09 (HCQ + AZ) compared to no HCQ). The study thus showed evidence of an association between increased mortality and HCQ in this cohort of COVID-19 patients but no change in rates of mechanical ventilation among the treatment conditions. The study had a few limitations: it was not randomized, and the baseline vital signs, laboratory tests, and prescription drug use were significantly different among the three groups. All of these factors could potentially influence treatment outcome. Furthermore, the authors acknowledge that the effect of the drugs might be different in females and pediatric subjects, since these subjects were not part of the study. The reported result that HCQ + AZ is safer than HCQ contradicts the findings of the previous large-scale analysis of twenty years of records that found HCQ + AZ to be more frequently associated with cardiac arrhythmia than HCQ alone [128]; whether this discrepancy is caused by the pathology of COVID-19, is influenced by age or sex, or is a statistical artifact is not presently known.

Finally, findings from the RECOVERY trial were released on October 8, 2020. This study used a randomized, open-label design to study the effects of HCQ compared to SOC at 176 hospitals in the United Kingdom [135]. This large study enrolled 11,197 hospitalized patients whose physicians believed it would not harm them to participate. Patients were randomized into either the control group or one of the treatment arms, with twice as many patients enrolled in the control group as any treatment group. Of the patients eligible to receive HCQ, 1,561 were randomized into the HCQ arm, and 3,155 were randomized into the control arm. The demographics of the HCQ and control groups were similar in terms of average age (65 years), proportion female (approximately 38%), ethnic make-up (73% versus 76% white), and prevalence of pre-existing conditions (56% versus 57% overall). In the HCQ arm of the study, patients received 800 mg at baseline and again after 6 hours, then 400 mg at 12 hours and every subsequent 12 hours. The primary outcome analyzed was all-cause mortality, and patient vital statistics were reported by physicians upon discharge or death, or else at 28 days following HCQ administration if they remained hospitalized. The secondary outcome assessed was the combined risk of progression to invasive mechanical ventilation or death within 28 days. By the advice of an external data monitoring committee, the HCQ arm of the study was reviewed early, leading to it being closed due a lack of support for HCQ as a treatment for COVID-19. The rates of COVID-19-related mortality reported in the RECOVERY trial did not differ between the control and HCQ arms (rate ratio, 1.09; 95% CI, 0.97 to 1.23; *p* = 0.15), but patients receiving HCQ were slightly more likely to die due to cardiac events (0.4 percentage points). Patients who received HCQ also had a longer duration of hospitalization than patients receiving usual care, being less likely to be discharged alive within 28 days (rate ratio 0.90; 95% CI, 0.83 to 0.98), and were more likely to progress to mechanical ventilation or death (risk ratio 1.14; 95% CI, 1.03 to 1.27). This large-scale study thus builds upon studies in the United States and China to suggest that HCQ is not an effective treatment, and in fact may negatively impact COVID-19 patients due to its side effects. Therefore, though none of the studies have been blinded, examining them together makes it clear that the available evidence points to significant dangers associated with the administration of HCQ to hospitalized COVID-19 patients, without providing any support for its efficacy.

#### HCQ for the Treatment of Mild Cases

One additional possible therapeutic application of HCQ considered was the treatment of mild COVID-19 cases in otherwise healthy individuals. This possibility was assessed in a randomized, open-label, multi-center analysis conducted in Catalonia (Spain) [136]. This analysis enrolled adults 18 and older who had been experiencing mild symptoms of COVID-19 for fewer than five days. Participants were randomized into an HCQ arm (N=136) and a control arm (N=157), and those in the treatment arm were administered 800 mg of HCQ on the first day of treatment followed by 400 mg on each of the subsequent six days. The primary outcome assessed was viral clearance at days 3 and 7 following the onset of treatment, and secondary outcomes were clinical progression and time to complete resolution of symptoms. No significant differences between the two groups were found: the difference in viral load between the HCQ and control groups was 0.01 (95% CI, −0.28 to 0.29) at day 3 and −0.07 (95% CI −0.44 to 0.29) at day 7, the relative risk of hospitalization was 0.75 (95% CI, 0.32 to 1.77), and the difference in time to complete resolution of symptoms was −2 days (*p* = 0.38). This study thus suggests that HCQ does not improve recovery from COVID-19, even in otherwise healthy adult patients with mild symptoms.

#### Prophylactic Administration of HCQ

An initial study of the possible prophylactic application of HCQ utilized a randomized, double-blind, placebo-controlled design to analyze the administration of HCQ prophylactically [137]. Asymptomatic adults in the United States and Canada who had been exposed to SARS-CoV-2 within the past four days were enrolled in an online study to evaluate whether administration of HCQ over five days influenced the probability of developing COVID-19 symptoms over a 14-day period. Of the participants, 414 received HCQ and 407 received a placebo. No significant difference in the rate of symptomatic illness was observed between the two groups (11.8% HCQ, 14.3% placebo, *p* = 0.35). The HCQ condition was associated with side effects, with 40.1% of patients reporting side effects compared to 16.8% in the control group (*p* < 0.001). However, likely due to the high enrollment of healthcare workers (66% of participants) and the well-known side effects associated with HCQ, a large number of participants were able to correctly identify whether they were receiving HCQ or a placebo (46.5% and 35.7%, respectively). Furthermore, due to a lack of availability of diagnostic testing, only 20 of the 107 cases were confirmed with a PCR-based test to be positive for SARS-CoV-2. The rest were categorized as “probable” or “possible” cases by a panel of four physicians who were blind to the treatment status. One possible confounder is that a patient presenting one or more symptoms, which included diarrhea, was defined as a “possible” case, but diarrhea is also a common side effect of HCQ. Additionally, four of the twenty PCR-confirmed cases did not develop symptoms until after the observation period had completed, suggesting that the 14-day trial period may not have been long enough or that some participants also encountered secondary exposure events. Finally, in addition to the young age of the participants in this study, which ranged from 32 to 51, there were possible impediments to generalization introduced by the selection process, as 2,237 patients who were eligible but had already developed symptoms by day 4 were enrolled in a separate study. It is therefore likely that asymptomatic cases were over-represented in this sample, which would not have been detected based on the diagnostic criteria used. Therefore, while this study does represent the first effort to conduct a randomized, double-blind, placebo-controlled investigation of HCQ’s effect on COVID-19 prevention after SARS-CoV-2 exposure in a large sample, the lack of PCR tests and several other design flaws significantly impede interpretation of the results. However, in line with the results from therapeutic studies, once again no evidence was found suggesting an effect of HCQ against COVID-19.

A second study [138] examined the effect of administering HCQ to healthcare workers as a pre-exposure prophylactic. The primary outcome assessed was the conversion from SARS-CoV-2 negative to SARS-CoV-2 positive status over the 8 week study period. This study was also randomized, double-blind, and placebo-controlled, and it sought to address some of the limitations of the first prophylactic study. The goal was to enroll 200 healthcare workers, preferentially those working with COVID-19 patients, at two hospitals within the University of Pennsylvania hospital system in Philadelphia, PA. Participants were randomized 1:1 to receive either 600 mg of HCQ daily or a placebo, and their SARS-CoV-2 infection status and antibody status were assessed using RT-PCR and serological testing, respectively, at baseline, 4 weeks, and 8 weeks following the beginning of the treatment period. The statistical design of the study accounted for interim analyses at 50 and 100 participants in case efficacy or futility of HCQ for prophylaxis became clear earlier than completion of enrollment. The 139 individuals enrolled comprised a study population that was fairly young (average age 33) and made of largely of people who were white, women, and without pre-existing conditions. At the second interim analysis, more individuals in the treatment group than the control group had contracted COVID-19 (4 versus 3), causing the estimated z-score to fall below the pre-established threshold for futility. As a result, the trial was terminated early, offering additional evidence against the use of HCQ for prophylaxis.

#### Summary of HCQ/CQ Research Findings

Early *in vitro* evidence indicated that HCQ could be an effective therapeutic against SARS-CoV-2 and COVID-19, leading to significant media attention and public interest in its potential as both a therapeutic and prophylactic. Initially it was hypothesized that CQ/HCQ might be effective against SARS-CoV-2 in part because CQ and HCQ have both been found to inhibit the expression of CD154 in T-cells and to reduce TLR signaling that leads to the production of pro-inflammatory cytokines [139]. Clinical trials for COVID-19 have more often used HCQ rather than CQ because it offers the advantages of being cheaper and having fewer side effects than CQ. However, research has not found support for a positive effect of HCQ on COVID-19 patients. Multiple clinical studies have already been carried out to assess HCQ as a therapeutic agent for COVID-19, and many more are in progress. To date, none of these studies have used randomized, double-blind, placebo-controlled designs with a large sample size, which would be the gold standard. Despite the design limitations (which would be more likely to produce false positives than false negatives), initial optimism about HCQ has largely dissipated. The most methodologically rigorous analysis of HCQ as a prophylactic [137] found no significant differences between the treatment and control groups, and the WHO’s global Solidarity trial similarly reported no effect of HCQ on mortality [31]. Thus, HCQ/CQ are not likely to be effective therapeutic or prophylactic agents against COVID-19. Additionally, one study identified an increased risk of mortality in older men receiving HCQ, and administration of HCQ and HCQ + AZ did not decrease the use of mechanical ventilation in these patients [134]. HCQ use for COVID-19 could also lead to shortages for anti-malarial or anti-rheumatic use, where it has documented efficacy. Despite significant early attention, these drugs appear to be ineffective against COVID-19. Several countries have now removed CQ/HCQ from their SOC for COVID-19 due to the lack of evidence of efficacy and the frequency of adverse effects.

#### Dexamethasone

Dexamethasone (9α-fluoro-16α-methylprednisolone) is a synthetic corticosteroid that binds to glucocorticoid receptors [140,141]. It was first synthesized in the late 1950s as an anti-inflammatory and has been used to treat RA and other inflammatory conditions [142,143], including allergies and asthma [144]. Steroids such as dexamethasone are widely available and affordable, and they are often used to treat community-acquired pneumonia [145]. A clinical trial that began in 2012 recently reported that dexamethasone may improve outcomes for patients with ARDS [146]. However, a meta-analysis of a small amount of available data about dexamethasone as a treatment for SARS suggested that it may, in fact, be associated with patient harm [147]; these findings may have been biased by the fact that all of the studies examined were observational and a large number of inconclusive studies were not included [148].

Dexamethasone works as an anti-inflammatory agent by binding to glucocorticoid receptors with higher affinity than endogenous cortisol [149]. In order to understand how dexamethasone reduces inflammation, it is necessary to consider the stress response broadly. In response to stress, corticotropin-releasing hormone stimulates the release of neurotransmitters known as catecholamines, such as epinephrine, and steroid hormones known as glucocorticoids, such as cortisol [150,151]. While catecholamines are often associated with the fight-or-flight response, the specific role that glucocorticoids play is less clear, although they are thought to be important to restoring homeostasis [152]. Immune challenge is a stressor that is known to interact closely with the stress response. The immune system can therefore interact with the central nervous system; for example, macrophages can both respond to and produce catecholamines [150]. Additionally, the production of both catecholamines and glucocorticoids is associated with inhibition of proinflammatory cytokines such as IL-6, IL-12, and tumor necrosis factor-α (TNF α) and the stimulation of anti-inflammatory cytokines such as IL-10, meaning that the stress response can regulate inflammatory immune activity [151]. Administration of dexamethasone has been found to correspond to dose-dependent inhibition of IL-12 production, but not to affect IL-10 [153]; the fact that this relationship could be disrupted by administration of a glucocorticoid-receptor antagonist suggests that it is regulated by the receptor itself [153]. Thus, the administration of dexamethasone for COVID-19 is likely to simulate the release of glucocorticoids endogenously during stress, resulting in binding of the synthetic steroid to the glucocorticoid receptor and the associated inhibition of the production of proinflammatory cytokines. In this model, dexamethasone reduces inflammation by stimulating the biological mechanism that reduces inflammation following a threat such as immune challenge.

Immunosuppressive drugs such as steroids are typically contraindicated in the setting of infection [154], but because COVID-19 results in hyperinflammation that appears to contribute to mortality via lung damage, immunosuppression may be a helpful approach to treatment [155]. The decision of whether and/or when to counter hyperinflammation with immunosuppression in the setting of COVID-19 was an area of intense debate, as the risks of inhibiting antiviral immunity needed to be weighed against the beneficial anti-inflammatory effects [156]. As a result, guidelines early in the pandemic typically recommended avoiding treating COVID-19 patients with corticosteroids such as dexamethasone [147].

The application of dexamethasone for the treatment of COVID-19 was evaluated as part of the multi-site RECOVERY trial in the United Kingdom [157]. Over 6,000 hospitalized COVID-19 patients were assigned into the SOC or treatment (dexamethasone) arms of the trial with a 2:1 ratio. At the time of randomization, some patients were ventilated (16%), others were on non-invasive oxygen (60%), and others were breathing independently (24%). Patients in the treatment arm were administered dexamethasone either orally or intravenously at 6 mg per day for up to 10 days. The primary end-point was the patient’s status at 28-days post-randomization (mortality, discharge, or continued hospitalization), and secondary outcomes analyzed included the progression to invasive mechanical ventilation over the same period. The 28-day mortality rate was found to be lower in the treatment group than in the SOC group (21.6% vs 24.6%, *p* < 0.001). However, this finding was driven by differences in mortality among patients who were receiving mechanical ventilation or supplementary oxygen at the start of the study. The report indicated that dexamethasone reduced 28-day mortality relative to SOC in patients who were ventilated (29.3% versus 41.4%) and among those who were receiving oxygen supplementation (23.3% versus 26.2%) at randomization, but not in patients who were breathing independently (17.8% versus 14.0%). One possible confounder is that patients receiving mechanical ventilation tended to be younger than patients who were not receiving respiratory support (by 10 years on average) and to have had symptoms for a longer period. However, adjusting for age did not change the conclusions, although the duration of symptoms was found to be significantly associated with the effect of dexamethasone administration. These findings also suggested that dexamethasone may have reduced progression to mechanical ventilation, especially among patients who were receiving oxygen support at randomization. Thus, this large, randomized, and multi-site, albeit not placebo-controlled, study suggests that administration of dexamethasone to patients who are unable to breathe independently may significantly improve survival outcomes. Additionally, dexamethasone is a widely available and affordable medication, raising the hope that it could be made available to COVID-19 patients globally.

The results of the RECOVERY trial’s analysis of dexamethasone suggest that this therapeutic is effective primarily in patients who had been experiencing symptoms for at least seven days and patients who were not breathing independently [158]. A meta-analysis that evaluated the results of the RECOVERY trial alongside trials of other corticosteroids, such as hydrocortisone, similarly concluded that corticosteroids may be beneficial to patients with severe COVID-19 who are receiving oxygen supplementation [159]. Thus, it seems likely that dexamethasone is useful for treating inflammation associated with immunopathy or cytokine release syndrome (CRS), which is a condition caused by detrimental overactivation of the immune system [1]. In fact, corticosteroids such as dexamethasone are sometimes used to treat CRS [160]. It is not surprising that administration of an immunosuppressant would be most beneficial when the immune system was dysregulated towards inflammation. However, it is also unsurprising that care must be taken in administering an immunosuppressant to patients fighting a viral infection. In particular, the concern has been raised that treatment with dexamethasone might increase patient susceptibility to concurrent (e.g., nosocomial) infections [161]. Additionally, the drug could potentially slow viral clearance and inhibit patients’ ability to develop antibodies to SARS-CoV-2 [147,161], with the lack of data about viral clearance being put forward as a major limitation of the RECOVERY trial [162]. Furthermore, dexamethasone has been associated with side effects that include psychosis, glucocorticoid-induced diabetes, and avascular necrosis [147], and the RECOVERY trial did not report outcomes with enough detail to be able to determine whether they observed similar complications. The effects of dexamethasone have also been found to differ among populations, especially in high-income versus middle- or low-income countries [163]. However, since the RECOVERY trial’s results were released, strategies have been proposed for administering dexamethasone alongside more targeted treatments to minimize the likelihood of negative side effects [161]. Given the available evidence, dexamethasone is currently the most promising treatment for severe COVID-19.

## Biologics

Biologics are produced from components of living organisms or viruses. They include treatments such as humanized monoclonal antibodies, tocilizumab (TCZ), and neutralizing antibodies (nAbs), and can also include prophylactics such as vaccines [4]. Historically produced from animal tissue, biologics have become increasingly feasible to produce as recombinant DNA technologies have advanced [164]. Often, they are glycoproteins or peptides [165], but whole viruses can also be used therapeutically or prophylactically, not only for vaccines but also as vectors for gene therapy or therapeutic proteins or for oncolytic virotherapy [166]. They are typically catabolized by the body to their amino acid components [165]. There are many differences on the development side between biologics and synthesized pharmaceuticals, such as small molecule drugs. Biologics are typically orders of magnitude larger than small molecule drugs, and their physiochemical properties are often much less understood [165]. They are often heat sensitive, and their toxicity can vary, as it is not directly associated with the primary effects of the drug [165]. However, this class includes some extremely significant medical breakthroughs, including insulin for the management of diabetes and the smallpox vaccine. As a result, biologics are another possible avenue through which the pharmacological management of SARS-CoV-2 infection can be approached.

### Tocilizumab

TCZ is a receptor antibody that was developed to manage chronic inflammation caused by the continuous synthesis of the cytokine IL-6 [167]. IL-6 is a pro-inflammatory cytokine belonging to the interleukin family, which is comprised by immune system regulators that are primarily responsible for immune cell differentiation. Often used to treat conditions such as RA [167], TCZ has become a pharmaceutical of interest for the treatment of COVID-19 because of the role IL-6 plays in this disease. It has also been approved to treat CRS caused by CAR-T treatments [168]. While secretion of IL-6 can be associated with chronic conditions, it is a key player in the innate immune response and is secreted by macrophages in response to the detection of pathogen-associated molecular patterns and damage-associated molecular patterns [167]. An analysis of 191 in-patients at two Wuhan hospitals revealed that blood concentrations of IL-6 differed between patients who did and did not recover from COVID-19. Patients who ultimately died had higher IL-6 levels at admission than those who recovered [169]. Additionally, IL-6 levels remained higher throughout the course of hospitalization in the patients who ultimately died [169]. This finding provided some early evidence that COVID-19 deaths may be induced by the hyperactive immune response, often referred to as CRS or cytokine storm syndrome (CSS), as IL-6 plays a key role in this response [170]. In this context, the observation of elevated IL-6 in patients who died may reflect an over-production of proinflammatory interleukins, suggesting that TCZ could potentially palliate some of the most severe symptoms of COVID-19 associated with increased cytokine production.

Human IL-6 is a 26-kDa glycoprotein that consists of 184 amino acids and contains two potential N-glycosylation sites and four cysteine residues. It binds to a type I cytokine receptor (IL-6Rα or glycoprotein 80) that exists in both membrane-bound (IL-6Rα) and soluble (sIL-6Rα) forms [171]. It is not the binding of IL-6 to the receptor that initiates pro- and/or anti-inflammatory signaling, but rather the binding of the complex to another subunit, known as IL-6Rβ or glycoprotein 130 (gp130) [171,172]. Unlike membrane-bound IL-6Rα, which is only found on hepatocytes and some types of leukocytes, gp130 is found on most cells [173]. When IL-6 binds to sIL-6Rα, the complex can then bind to a gp130 protein on any cell [173]. The binding of IL-6 to IL-6Rα is termed classical signaling, while its binding to sIL-6Rα is termed trans-signaling [173,174,175]. These two signaling processes are thought to play different roles in health and illness. For example, trans-signaling may play a role in the proliferation of mucosal T-helper TH2 cells associated with asthma, while an earlier step in this proliferation process may be regulated by classical signaling [173]. Similarly, IL-6 is known to play a role in Crohn’s Disease via trans-, but not classical, signaling [173]. Both classical and trans-signaling can occur through three independent pathways: the Janus-activated kinase-STAT3 pathway, the Ras/Mitogen-Activated Protein Kinases pathway and the Phosphoinositol-3 Kinase/Akt pathway [171]. These signaling pathways are involved in a variety of different functions, including cell type differentiation, immunoglobulin synthesis, and cellular survival signaling pathways, respectively [171]. The ultimate result of the IL-6 cascade is to direct transcriptional activity of various promoters of pro-inflammatory cytokines, such as IL-1, TFN, and even IL-6 itself, through the activity of NF-κB [171]. IL-6 synthesis is tightly regulated both transcriptionally and post-transcriptionally, and it has been shown that viral proteins can enhance transcription of the IL-6 gene by strengthening the DNA-binding activity between several transcription factors and IL-6 gene-cis-regulatory elements [176]. Therefore, drugs inhibiting the binding of IL-6 to IL-6Rα or sIL-6Rα are of interest for combating the hyperactive inflammatory response characteristic of CRS/CSS. TCZ is a humanized monoclonal antibody that binds both to the insoluble and soluble receptor of IL-6, providing de facto inhibition of the IL-6 immune cascade.

Tocilizumab is being administered either as an intervention or as concomitant medication in 77 interventional COVID-19 clinical trials (Figure 2). No randomized, placebo-controlled studies of TCZ have currently released results. Therefore, no conclusions can be drawn about its efficacy for the treatment of COVID-19. However, early interest in TCZ as a possible treatment for COVID-19 emerged from a very small retrospective study in China that examined 20 patients with severe symptoms in early February 2020 and reported rapid improvement in symptoms following treatment with TCZ [177]. Subsequently, a number of retrospective studies have been conducted in several countries. Many studies use a retrospective, observational design, where they compare outcomes for COVID-19 patients who received TCZ to those who did not over a set period of time. For example, one of the largest retrospective, observational analyses released to date [178], consisting of 1,351 patients admitted to several care centers in Italy, compared the rates at which patients who received TCZ died or progressed to invasive medical ventilation over a 14-day period compared to patients receiving only SOC. Under this definition, SOC could include other drugs such as HCQ, azithromycin, lopinavir-ritonavir or darunavir-cobicistat, or heparin. While this study was not randomized, a subset of patients who were eligible to receive TCZ were unable to obtain it due to shortages; however, these groups were not directly compared in the analysis. After adjusting for variables such as age, sex, and SOFA (sequential organ failure assessment) score, they found that patients treated with TCZ were less likely to progress to invasive medical ventilation and/or death (adjusted HR = 0.61, CI 0.40–0.92, *p* = 0.020); analysis of death and ventilation separately suggests that this effect may have been driven by differences in the death rate (20% of control versus 7% of TCZ-treated patients). The study reported particular benefits for patients whose PaO_2_/FiO_2_ ratio, also known as the Horowitz Index for Lung Function, fell below a 150 mm Hg threshold. They found no differences between groups administered subcutaneous versus intravenous TCZ.

Another retrospective observational analysis of interest examined the charts of patients at a hospital in Connecticut, USA where 64% of all 239 COVID-19 patients in the study period were administered TCZ based on assignment by a standardized algorithm [179]. They found that TCZ administration was associated with more similar rates of survivorship in patients with severe versus nonsevere COVID-19 at intake, defined based on the amount of supplemental oxygen needed. They therefore proposed that their algorithm was able to identify patients presenting with or likely to develop CRS as good candidates for TCZ. This study also reported higher survivorship in Black and Hispanic patients compared to white patients when adjusted for age. The major limitation with interpretation for these studies is that there may be clinical characteristics that influenced medical practitioners decisions to administer TCZ to some patients and not others. One interesting example therefore comes from an analysis of patients at a single hospital in Brescia, Italy, where TCZ was not available for a period of time [180]. This study compared COVID-19 patients admitted to the hospital before and after March 13, 2020, when the hospital received TCZ. Therefore, patients who would have been eligible for TCZ prior to this arbitrary date did not receive it as treatment, making this retrospective analysis something of a natural experiment. Despite this design, demographic factors did not appear to be consistent between the two groups, and the average age of the control group was older than the TCZ group. The control group also had a higher percentage of males and a higher incidence of comorbidities such as diabetes and heart disease. All the same, the multivariate HR, which adjusted for these clinical and demographic factors, found a significant difference between survival in the two groups (HR=0.035, CI=0.004–0.347, *p* = 0.004). The study reported improvement of survival outcomes after the addition of TCZ to the SOC regime, with 11 of 23 patients (47.8%) admitted prior to March 13th dying compared to 2 of 62 (3.2%) admitted afterwards (HR=0.035; 95% CI, 0.004 to 0.347; *p* = 0.004). They also reported a reduced progression to mechanical ventilation in the TCZ group. However, this study also holds a significant limitation: the time delay between the two groups means that knowledge about how to treat the disease likely improved over this timeframe as well. All the same, the results of these observational retrospective studies provide support for TCZ as a pharmaceutical of interest for follow-up in clinical trials.

Other retrospective analyses have utilized a case-control design to match pairs of patients with similar baseline characteristics, only one of whom received TCZ for COVID-19. In one such study, TCZ was significantly associated with a reduced risk of progression to ICU admission or death [181]. This study examined only 20 patients treated with TCZ (all but one of the patients treated with TCZ in the hospital during the study period) and compared them to 25 patients receiving SOC. For the combined primary endpoint of death and/or ICU admission, only 25% of patients receiving TCZ progressed to an endpoint compared to 72% in the SOC group (*p* = 0.002, presumably based on a chi-square test based on the information provided in the text). When the two endpoints were examined separately, progression to invasive medical ventilation remained significant (32% SOC compared to 0% TCZ, *p* = 0.006) but not for mortality (48% SOC compared to 25% TCZ, *p* = 0.066). In contrast, a study that compared 96 patients treated with TCZ to 97 patients treated with SOC only in New York City found that differences in mortality did not differ between the two groups, but that this difference did become significant when intubated patients were excluded from the analysis [182]. Taken together, these findings suggest that future clinical trials of TCZ may want to include intubation as an endpoint. However, these studies should be approached with caution, not only because of the small number of patients enrolled and the retrospective design, but also because they performed a large number of statistical tests and did not account for multiple hypothesis testing. In general, caution must be exercised when interpreting subgroup analyses after a primary combined endpoint analysis. These last findings highlight the need to search for a balance between impairing a harmful immune response, such as the one generated during CRS/CSS, and preventing the worsening of the clinical picture of the patients by potential new viral infections. Though data about TCZ for COVID-19 is still only just emerging, some meta-analyses and systematic reviews have investigated the available data. One meta-analysis [183] evaluated 19 studies published or released as preprints prior to July 1, 2020 and found that the overall trends were supportive of the frequent conclusion that TCZ does improve survivorship, with a significant HR of 0.41 (*p* < 0.001). This trend improved when they excluded studies that administered a steroid alongside TCZ, with a significant HR of 0.04 (*p* < 0.001). They also found some evidence for reduced invasive ventilation or ICU admission, but only when excluding all studies except a small number whose estimates were adjusted for the possible bias introduced by the challenges of stringency during the enrollment process. A systematic analysis of sixteen case-control studies of TCZ estimated an odds ratio of mortality of 0.453 (95% CI 0.376–0.547, *p* < 0.001), suggesting possible benefits associated with TCZ treatment [184]. Although these estimates are similar, it is important to note that they are drawing from the same literature and are therefore likely to be affected by the same potential biases in publication. A different systematic review of studies investigating TCZ treatment for COVID-19 analyzed 31 studies that had been published or released as pre-prints and reported that none carried a low risk of bias [185]. Therefore, the present evidence is not likely to be sufficient for conclusions about the efficacy of TCZ.

On February 11, 2021, a preprint describing the first randomized control trial of TCZ was released as part of the RECOVERY trial [186]. Of the 21,550 patients enrolled in the RECOVERY trial at the time, 4,116 adults hospitalized with COVID-19 across the 131 sites in the United Kingdom were assigned to the arm of the trial evaluating the effect of TCZ. Among them, 2,022 were randomized to receive TCZ and 2,094 were randomized to SOC, with 79% of patients in each group available for analysis at the time that the initial report was released. The primary outcome measured was 28-day mortality, and TCZ was found to reduce 28-day mortality from 33% of patients receiving SOC alone to 29% of those receiving TCZ, corresponding to a rate ratio of 0.86 (95% CI 0.77–0.96; *p* = 0.007). TCZ was also significantly associated with the probability of hospital discharge within 28 days for living patients, which was 47% in the SOC group and 54% in the TCZ group (rate ratio 1.22, 95% CI 1.12–1.34, *p* < 0.0001). A potential statistical interaction between TCZ and corticosteroids was observed, with the combination providing greater mortality benefits than TCZ alone, but the authors note that caution is advisable in light of the number of statistical tests conducted. Combining the RECOVERY trial data with data from seven smaller randomized control trials indicates that TCZ is associated with a 13% reduction in 28-day mortality (rate ratio 0.87, 95% CI 0.79–0.96, *p* = 0·005) [186]. While this initial report did not include the full results expected from the RECOVERY trial, this large-scale, randomized controlled trial therefore offers strong evidence that TCZ may offer benefits for COVID-19 patients, even at this initial stage of analysis.

There are possible risks associated with the administration of TCZ for COVID-19. TCZ has been used for over a decade to treat RA [187], and a recent study found the drug to be safe for pregnant and breastfeeding women [188]. However, TCZ may increase the risk of developing infections [187], and RA patients with chronic hepatitis B infections had a high risk of hepatitis B virus reactivation when TCZ was administered in combination with other RA drugs [189]. As a result, TCZ is contraindicated in patients with active infections such as tuberculosis [190]. Previous studies have investigated, with varying results, a possible increased risk of infection in RA patients administered TCZ [191,192], although another study reported that the incidence rate of infections was higher in clinical practice RA patients treated with TCZ than in the rates reported by clinical trials [193]. In the investigation of 544 Italian COVID-19 patients, the group treated with TCZ was found to be more likely to develop secondary infections, with 24% compared to 4% in the control group (*p* < 0.0001) [178]. Reactivation of hepatitis B and herpes simplex virus 1 was also reported in a small number of patients in this study, all of whom were receiving TCZ. A July 2020 case report described negative outcomes of two COVID-19 patients after receiving TCZ, including one death; however, both patients were intubated and had entered septic shock prior to receiving TCZ [194], likely indicating a severe level of cytokine production. Additionally, D-dimer and sIL2R levels were reported by one study to increase in patients treated with TCZ, which raised concerns because of the potential association between elevated D-dimer levels and thrombosis and between sIL2R and diseases where T-cell regulation is compromised [179]. An increased risk of bacterial infection was also identified in a systematic review of the literature, based on the unadjusted estimates reported [183]. In the RECOVERY trial, however, only three out of 2,022 participants in the group receiving TCZ developed adverse reactions determined to be associated with the intervention, and no excess deaths were reported [186]. TCZ administration to COVID-19 patients is not without risks and may introduce additional risk of developing secondary infections; however, while caution may be prudent when treating patients who have latent viral infections, the results of the RECOVERY trial indicate that adverse reactions to TCZ are very rare among COVID-19 patients broadly.

In summary, approximately 33% of hospitalized COVID-19 patients develop ARDS [195], which is caused by an excessive early response of the immune system which can be a component of CRS/CSS [179,190]. This overwhelming inflammation is triggered by IL-6. TCZ is an inhibitor of IL-6 and therefore may neutralize the inflammatory pathway that leads to the cytokine storm. While the mechanism suggests TCZ could be beneficial for the treatment of COVID-19 patients experiencing excessive immune activity, no randomized controlled trials are available assessing its effect. However, small initial studies have found preliminary indications that TCZ may reduce progression to invasive medical ventilation and/or death. It should be noted that SOC varied widely across retrospective studies, with one study administering HCQ, lopinavir-ritonavir, antibiotics, and/or heparin as part of SOC. Interest in TCZ as a treatment for COVID-19 was supported by two meta-analyses [183,196], but a third meta-analysis found that all of the available literature carries a risk of bias, with even the largest available TCZ studies to date carrying a moderate risk of bias under the ROBINS-I criteria [185]. Additionally, different studies used different dosages, number of doses, and methods of administration. Ongoing research may be needed to optimize administration of TCZ [197], although similar results were reported by one study for intravenous and subcutaneous administration [178]. Clinical trials that are in progress are likely to provide additional insight into the effectiveness of this drug for the treatment of COVID-19 along with how it should be administered.

### Monoclonal Neutralizing Antibodies

Monoclonal antibodies have revolutionized the way we treat human diseases. They have become some of the best-selling drugs in the pharmaceutical market in recent years [198]. There are currently 79 FDA approved mAbs on the market, including antibodies for viral infections (e.g. Ibalizumab for HIV and Palivizumab for RSV) [198,199]. Virus-specific neutralizing antibodies commonly target viral surface glycoproteins or host structures, thereby inhibiting viral entry through receptor binding interference [200,201]. This is predicted to reduce the viral load, mitigate disease, and reduce overall hospitalization. While polyclonal antibodies from convalescent plasma can be used as a treatment for COVID-19, this section focuses on current efforts in developing monoclonal neutralizing antibodies (nAbs) against SARS-CoV-2 (excellent reviews regarding convalescent plasma therapy can be found here [202,203]). Specifically, we focus on monoclonal antibodies that have recently been granted emergency use authorization and discuss the challenges in the successful development of monoclonal neutralizing antibodies.

During the first SARS epidemic in 2002, nAbs were found in SARS-CoV-1-infected patients [204,205]. Several studies following up on these findings identified various S-glycoprotein epitopes as the major targets of nAbs against SARS-CoV-1 [206]. Coronaviruses use trimeric spike (S) glycoproteins on their surface to bind to the host cell, allowing for cell entry [66,207]. Each S glycoprotein protomer is comprised of an S1 domain, also called the RBD, and an S2 domain. The S1 domain binds to the host cell while the S2 domain facilitates the fusion between the viral envelope and host cell membranes [206]. The genomic identity between the RBD of SARS-CoV-1 and SARS-CoV-2 is around 74% [208]. Due to this high degree of similarity, preexisting antibodies against SARS-CoV-1 were initially considered candidates for neutralizing activity against SARS-CoV-2. While some antibodies developed against the SARS-CoV-1 spike protein showed cross-neutralization activity with SARS-CoV-2 [209,210], others failed to bind to SARS-CoV-2 spike protein at relevant concentrations [211]. Cross-neutralizing activities were dependent on whether the epitope recognized by the antibodies were conserved between SARS-CoV-1 and SARS-CoV-2 [209].

The first human monoclonal neutralizing antibody specifically against the SARS-CoV-2 S glycoprotein was developed using hybridoma technology [212], where antibody-producing B-cells developed by mice are inserted into myeloma cells to produce a hybrid cell line (the hybridoma) that is grown in culture. The 47D11 antibody clone was able to cross-neutralize SARS-CoV-1 and SARS-CoV-2. This antibody (now ABVV-47D11) has recently entered clinical trials in collaboration with AbbVie. Since then, an extensive monoclonal neutralizing antibody pipeline has been developed to combat the ongoing pandemic, with over 50 different antibodies in clinical trials [213] and two treatments recently receiving emergency use authorization by the FDA.

#### Bamlanivimab (LY-CoV555) and Etesevimab (LY-CoV016)

Bamlanivimab is a human monoclonal antibody that was derived from convalescent plasma donated by recovered COVID-19 patient, evaluated in research by the National Institute of Allergy and Infectious Diseases (NIAID), and subsequently developed by AbCellera and Eli Lilly. The neutralizing activity of bamlanivimab was initially demonstrated *in vivo* using a nonhuman primate model [214]. In these studies, prophylactic Ly-CoV555 infusions protected rhesus macaques from SARS-CoV-2 infection. Based on the positive preclinical data, Eli Lilly initiated the first human clinical trial for a monoclonal antibody against SARS-CoV-2. The phase 1 trial, which was conducted in hospitalized COVID-19 patients, was completed in August 2020 [215].

Estesevimab (LY-CoV016 or JS-016) is also a monoclonal neutralizing antibody against the spike protein of SARS-CoV-2. It was initially developed by Junshi Biosciences and later licensed and developed through Eli Lilly. A phase 1 clinical trial to assess the safety of etesevimab was completed in October 2020 [216]. Etesevimab was shown to bind a different epitope on the spike protein than bamlanivimab, suggesting that the two antibodies used as a combination therapy would further enhance their clinical use compared to a monotherapy [217].

To assess the efficacy and safety of bamlanivimab alone or in combination with etesevimab for the treatment of COVID-19, a phase 2/3 trial (BLAZE-1) [218] was initiated. The interim analysis of the phase 2 portion suggested that bamlanivimab alone was able to reduce accelerate the reduction in viral load [219]. However, more recent data suggests that only the bamlanivimab/etesevimab combination therapy is able to reduce viral load in COVID-19 patients [217]. Based on this data, the combination therapy received emergency use authorization for COVID-19 from the FDA in February of 2021.

#### Casirivimab and Imdevimab (REGN-COV2)

Casirivimab (REGN10933) and imdevimab (REGN10987) are two monoclonal antibodies against the SARS-CoV-2 spike protein. They were both developed by Regeneron in a parallel high-throughput screen to identify neutralizing antibodies from either humanized mice or patient-derived convalescent plasma [220]. In these efforts, multiple antibodies were characterized for their ability to bind and neutralize the SARS-CoV-2 spike protein. The authors hypothesized that an antibody cocktail, rather than each individual antibody, could increase the therapeutic efficacy while minimizing the risk for virus escape. Therefore, the authors tested pairs of individual antibodies for their ability to simultaneously bind the RBD of the spike protein. Based on this data, casirivimab and imdevimab were identified as the lead antibody pair, resulting in the initiation of two clinical trials [221,222]. Data from this phase 1–3 trial published in the *New England Journal of Medicine* shows that the REGN-COV2 antibody cocktail reduced viral load, particularly in patients with high viral load or whose endogenous immune response had not yet been initiated [223]. However, in patients who already initiated an immune response, exogenous addition of REGN-COV2 did not improve the endogenous immune response. Both doses were well tolerated with no serious events related to the antibody cocktail. Based on this data, the FDA granted emergency use authorization for REGN-COV2 in patients with mild to moderate COVID-19 who are at risk to develop severe disease. Ongoing efforts are trying to evaluate the efficacy of REGN-COV2 to improve clinical outcomes in hospitalized patients [221].

#### Viral Resistance to Neutralizing Antibodies

With the ongoing global spread of new SARS-CoV-2 variants, there is a growing concern that mutations in SARS-CoV-2 spike protein could escape antibody neutralization, thereby reducing the efficacy of monoclonal antibody therapeutics and vaccines. A comprehensive mutagenesis screen recently identified several amino acid substitutions in the SARS-CoV-2 spike protein that can prevent antibody neutralization [224]. While some mutations result in resistance to only one antibody, others confer broad resistance to multiple monoclonal antibodies as well as polyclonal human sera, suggesting that some amino acids are “hotspots” for antibody resistance. However, it was not investigated whether the resistant mutations identified result in a fitness advantage. Accordingly, an impact on neutralizing efficiency has been reported for the emerging UK (B.1.1.7) and South Africa (B.1.351) variants [225,226,227]. While the reported impact on antibody neutralization needs to be confirmed *in vivo*, it suggests that some adjustments to therapeutic antibody treatments may be necessary to maintain the efficacy that was reported in previous clinical trials.

Antibody cocktails such as REGN-COV2, CT-P59, and AZD7442 have been developed to overcome the risk for attenuation of neutralizing activity of a single monoclonal antibody. These cocktails consist of antibodies that recognize different epitopes on the spike protein, decreasing the likelihood that a single amino acid change can cause resistance to all antibodies in the cocktail. However, neutralizing resistance can emerge even against an antibody cocktail if the individual antibodies target subdominant epitopes [226].

Another strategy is to develop broadly neutralizing antibodies that target structures that are highly conserved, as these are less likely to mutate [228,229] or to target epitopes that are insensitive to mutations [230]. One such antibody (ADG-2) has recently been reported [231]. This antibody targets a highly conserved epitope that overlaps the hACE2 binding site of all clade 1 sarbecoviruses. Prophylactic administration of ADG-2 in an immunocompetent mouse model of COVID-19 resulted in protection against viral replication in the lungs and respiratory burden. Since the epitope targeted by ADG-2 represents an Achilles’ heel for clade 1 sarbecoviruses, this antibody might be a promising candidate against all circulating variants as well as emerging SARS-related coronaviruses.

In just under a year since the structure of the SARS-CoV-2 spike protein was first published, an impressive pipeline of monoclonal antibodies targeting SARS-CoV-2 has entered clinical trials, with hundreds more candidates in preclinical stages. Technological advances in antibody drug design as well as in structural biology massively accelerated the discovery of novel antibody candidates and the mechanisms by which they interact with the target structure. One of the biggest challenges remains identifying antibodies that not only bind to their target, but also prove to be beneficial for disease management. Thus far, two antibody cocktails (REGN-COV2 and LY-CoV555/LY-COV016) have been granted emergency use authorization by the FDA. However, their current use is limited to people with mild to moderate disease that are not hospitalized. Therefore, it has yet to be determined whether monoclonal antibodies can be used as a successful treatment option for severe COVID patients.

### Interferons

IFNs are a family of cytokines critical to activating the innate immune response against viral infections. Interferons are classified into three categories based on their receptor specificity: types I, II and III [170]. Specifically, IFNs I (IFN-*α* and *β*) and II (IFN-*γ*) induce the expression of antiviral proteins [232]. Among these IFNs, IFN-*β* has already been found to strongly inhibit the replication of other coronaviruses, such as SARS-CoV-1, in cell culture, while IFN-*α* and *γ* were shown to be less effective in this context [232]. There is evidence that patients with higher susceptibility to ARDS indeed show deficiency in IFN-*β* For instance, infection with other coronaviruses impairs IFN-*β* expression and synthesis, allowing the virus to escape the innate immune response [233]. On March 18 2020, Synairgen plc received approval to start a phase II trial for SNG001, an IFN-*β*−1a formulation to be delivered to the lungs via inhalation [234]. SNG001, which contains recombinant interferon beta-1a, was previously shown to be effective in reducing viral load in an *in vivo* model of swine flu and *in vitro* models of other coronavirus infections [235]. In July, a press release from Synairgen stated that SNG001 reduced progression to ventilation in a double-blind, placebo-controlled, multi-center study of 101 patients with an average age in the late 50s [236]. These results were subsequently published in November 2020 [237]. The study reports that the participants were assigned at a ratio of 1:1 to receive either SNG001 or a placebo that lacked the active compound, by inhalation for up to 14 days. The primary outcome they assessed was the change in patients’ score on the WHO Ordinal Scale for Clinical Improvement (OSCI) at trial day 15 or 16. SNG001 was associated with an odds ratio of improvement on the OSCI scale of 2.32 (95% CI 1.07 – 5.04, *p* = 0.033) in the intention-to-treat analysis and 2.80 (95% CI 1.21 – 6.52, *p* = 0.017) in the per-protocol analysis, corresponding to significant improvement in the SNG001 group on the OSCI at day 15/16. Some of the secondary endpoints analyzed also showed differences: at day 28, the OR for clinical improvement on the OSCI was 3.15 (95% CI 1.39 – 7.14, *p* = 0.006), and the odds of recovery at day 15/16 and at day 28 were also significant between the two groups. Thus, this study suggested that IFN-*β*1 administered via SNG001 may improve clinical outcomes.

In contrast, the WHO Solidarity trial reported no significant effect of IFN-*β* 1a on patient survival during hospitalization [31]. Here, the primary outcome analyzed was in-hospital mortality, and the rate ratio for the two groups was 1.16 (95% CI, 0.96 to 1.39; *p* = 0.11) administering IFN-*β−*1a to 2050 patients and comparing their response to 2,050 controls. However, there are a few reasons that the different findings of the two trials might not speak to the underlying efficacy of this treatment strategy. One important consideration is the stage of COVID-19 infection analyzed in each study. The Synairgen trial enrolled only patients who were not receiving invasive ventilation, corresponding to a less severe stage of disease than many patients enrolled in the SOLIDARITY trial, as well as a lower overall rate of mortality [238]. Additionally, the methods of administration differed between the two trials, with the SOLIDARITY trial administering IFN-*β*−1a subcutaneously [238]. The differences in findings between the studies suggests that the method of administration might be relevant to outcomes, with nebulized IFN-*β*−1a more directly targeting receptors in the lungs. A trial that analyzed the effect of subcutaneously administered IFN-β−1a on patients with ARDS between 2015 and 2017 had also reported no effect on 28-day mortality [239], while a smaller study analyzing the effect of subcutaneous IFN administration did find a significant improvement in 28-day mortality for COVID-19 [240]. At present, several ongoing clinical trials are investigating the potential effects of IFN-*β*−1a, including in combination with therapeutics such as remdesivir [241] and administered via inhalation [234]. Thus, as additional information becomes available, a more detailed understanding of whether and under which circumstances IFN-*β*−1a is beneficial to COVID-19 patients should develop.

## Discussion

With the emergence of the COVID-19 pandemic caused by the coronavirus SARS-CoV-2, the development and identification of therapeutic and prophylactic interventions became issues of international urgency. In previous outbreaks of HCoV, namely SARS and MERS, the development of these interventions was very limited. As research has progressed, several potential approaches to treatment have emerged (Figure 3). Most notably, remdesivir has been approved by the FDA for the treatment of COVID-19, and dexamethasone, which was approved by the FDA in 1958, has been found to improve outcomes for patients with severe COVID-19. Other potential therapies are being still being explored and require additional data (Figure 2). As more evidence becomes available, the potential for existing and novel therapies to improve outcomes for COVID-19 patients will become better understood.

Insights into the pathogenesis of and immune response to SARS-CoV-2 (see [1]) have also guided the identification of potential prophylactics and therapeutics. As cases have become better characterized, it has become evident that many patients experience an initial immune response to the virus that is typically characterized by fever, cough, dyspnea, and related symptoms. However, the most serious concern is CRS, when the body’s immune response becomes dysregulated, resulting in an extreme inflammatory response. The RECOVERY trial, a large-scale, multi-arm trial enrolling about 15% of all COVID-19 patients in the United Kingdom, was the first to identify that the widely available steroid dexamethasone seems to be beneficial for patients suffering from this immune dysregulation [157]. The results of efforts to identify therapeutic treatments to treat patients early in the course of infection have been more ambiguous. Early interest in the drugs HCQ and CQ yielded no promising results from studies with robust experimental designs. On the other hand, the experimental drug remdesivir, which was developed as a candidate therapeutic for EVD, has received enough support from early analyses to receive FDA approval, although results have been mixed. The potential for other drugs, such as tocilizumab, to reduce recovery time remains unclear, but some early results were promising.

One additional concern is that the presentation of COVID-19 appears to be heterogeneous across the lifespan. Many adult cases, especially in younger adults, present with mild symptoms or even asymptomatically, while others, especially in older adults, can be severe or fatal. In children, the SARS-CoV-2 viral infection can present either as a respiratory illness comparable to COVID-19 or as an inflammatory condition, known as multisystem inflammatory syndrome in children, for which presentation is similar to Kawasaki Disease [242]. The therapeutics and prophylactics discussed here were primarily tested in adults, and additional research is needed to identify therapeutics that address the symptoms characteristic of pediatric COVID-19 and MIS-C cases.

### Potential Avenues of Interest for Therapeutic Development

Given what is currently known about these therapeutics for COVID-19, a number of related therapies beyond those explored above may also prove to be of interest. For example, the demonstrated benefit of dexamethasone and the ongoing potential of tocilizumab for treatment of COVID-19 suggests that other anti-inflammatory agents might also hold value for the treatment of COVID-19. Current evidence supporting the treatment of severe COVID-19 with dexamethasone suggests that the need to curtail the cytokine storm inflammatory response transcends the risks of immunosuppression, and other anti-inflammatory agents may therefore benefit patients in this phase of the disease. While dexamethasone is considered widely available and generally affordable, the high costs of biologics such as tocilizumab therapy may present obstacles to wide-scale distribution of this drug if it proves of value. At the doses used for RA patients, the cost for tocilizumab ranges from $179.20 to $896 per dose for the IV form and $355 for the pre-filled syringe [243]. Several other anti-inflammatory agents used for the treatment of autoimmune diseases may also be able to counter the effects of the cytokine storm induced by the virus, and some of these, such as cyclosporine, are likely to be more cost-effective and readily available than biologics [244]. While tocilizumab targets IL-6, several other inflammatory markers could be potential targets, including TNF-α. Inhibition of TNF-α by a compound such as Etanercept was previously suggested for treatment of SARS-CoV-1 [245] and may be relevant for SARS-CoV-2 as well. Another anti-IL-6 antibody, sarilumab, is also being investigated [246,247]. Baricitinib and other small molecule inhibitors of the Janus-activated kinase pathway also curtail the inflammatory response and have been suggested as potential options for SARS-CoV-2 infections [248]. Baricitinib, in particular, may be able to reduce the ability of SARS-CoV-2 to infect lung cells [249]. Clinical trials studying baricitinib in COVID-19 have already begun in the US and in Italy [250,251]. Identification and targeting of further inflammatory markers that are relevant in SARS-CoV-2 infection may be of value for curtailing the inflammatory response and lung damage.

In addition to immunosuppressive treatments, which are most beneficial late in disease progression, much research is focused on identifying therapeutics for early-stage patients. For example, although studies of HCQ have not supported the early theory-driven interest in this antiviral treatment, alternative compounds with related mechanisms may still have potential. Hydroxyferroquine derivatives of HCQ have been described as a class of bioorganometallic compounds that exert antiviral effects with some selectivity for SARS-CoV-1 *in vitro* [252]. Future work could explore whether such compounds exert antiviral effects against SARS-CoV-2 and whether they would be safer for use in COVID-19. Another potential approach is the development of antivirals, which could be broad-spectrum, specific to coronaviruses, or targeted to SARS-CoV-2. Development of new antivirals is complicated by the fact that none have yet been approved for human coronaviruses. Intriguing new options are emerging, however. Beta-D-N4-hydroxycytidine is an orally bioavailable ribonucleotide analog showing broad-spectrum activity against RNA viruses, which may inhibit SARS-CoV-2 replication *in vitro* and *in vivo* in mouse models of HCoVs [253]. A range of other antivirals are also in development. Development of antivirals will be further facilitated as research reveals more information about the interaction of SARS-CoV-2 with the host cell and host cell genome, mechanisms of viral replication, mechanisms of viral assembly, and mechanisms of viral release to other cells; this can allow researchers to target specific stages and structures of the viral life cycle. Finally, antibodies against viruses, also known as antiviral monoclonal antibodies, could be an alternative as well and are described in detail in an above section. The goal of antiviral antibodies is to neutralize viruses through either cell-killing activity or blocking of viral replication [254]. They may also engage the host immune response, encouraging the immune system to hone in on the virus. Given the cytokine storm that results from immune system activation in response to the virus, which has been implicated in worsening of the disease, an nAb may be preferable. Upcoming work may explore the specificity of nAbs for their target, mechanisms by which the nAbs impede the virus, and improvements to antibody structure that may enhance the ability of the antibody to block viral activity.

Some research is also investigating potential therapeutics and prophylactics that would interact with components of the innate immune response. For example, TLRs are pattern recognition receptors that recognize pathogen- and damage-associated molecular patterns and contribute to innate immune recognition and, more generally, promotion of both the innate and adaptive immune responses [255]. In mouse models, poly(I:C) and CpG, which are agonists of Toll-like receptors TLR3 and TLR9, respectively, showed protective effects when administered prior to SARS-CoV-1 infection [256]. Therefore, TLR agonists hold some potential for broad-spectrum prophylaxis.

Given that a large number of clinical trials are currently in progress, more information about the potential of these and other therapeutics should become available over time. This information, combined with advances in understanding the molecular structure and viral pathogenesis of SARS-CoV-2, may lead to a more complete understanding of how the virus affects the human host and what strategies can improve outcomes. To date, investigations of potential therapeutics for COVID-19 have focused primarily on repurposing existing drugs. This approach is necessary given the urgency of the situation as well as the extensive time required for developing and testing new therapies. However, in the long-term, new drugs specific for treatment of COVID-19 may also enter development. Development of novel drugs is likely to be guided by what is known about the pathogenesis and molecular structure of SARS-CoV-2. For example, understanding the various structural components of SARS-CoV-2 may allow for the development of small molecule inhibitors of those components. Currently, crystal structures of the SARS-CoV-2 main protease have recently been resolved [67,257], and efforts are already in place to perform screens for small molecule inhibitors of the main protease, which have yielded potential hits [67]. Much work remains to be done to determine further crystal structures of other viral components, understand the relative utility of targeting different viral components, perform additional small molecule inhibitor screens, and determine the safety and efficacy of the potential inhibitors. While still nascent, work in this area is promising. Over the longer term, this approach and others may lead to the development of novel therapeutics specifically for COVID-19 and SARS-CoV-2.

## Conclusions

Due to the large number of clinical trials currently under examination (Figure 2), not all candidates are examined here (Table 1). Instead, this review seeks to provide an overview of the range of mechanisms that have been explored and to examine some prominent candidates in the context of the pathogenesis of and immune response to SARS-CoV-2. As more research becomes available, this review will be updated to include additional therapeutics that emerge and to include new findings that are released about those discussed here. While no therapeutics or vaccines were developed for SARS-CoV-1 or MERS-CoV, the current state of COVID-19 research suggests that the body of literature produced before and after the emergence of these viruses has prepared the biomedical community for a rapid response to novel HCoV like SARS-CoV-2. As the COVID-19 pandemic continues to be a topic of significant worldwide concern, more information is expected to become available about pharmaceutical mechanisms that can be used to combat this, and possibly other, HCoV. These advances therefore not only benefit the international community’s ability to respond to the current crisis, but are also likely to shape responses to future viral threats.

## Figures and Tables

**Table 1:** Summary table of candidate therapeutics examined in this manuscript. The FDA status is provided where available. The evidence available is based on the progression of the therapeutic through the pharmaceutical development pipeline, with randomized control trials (RCT) as the most informative source of evidence. The effectiveness is summarized based on the current available evidence; large trials such as RECOVERY and Solidarity are weighted heavily in this summary. This table was last updated on February 17, 2021.

Treatment	Category	FDA Status	Evidence Available	Suggested Effectiveness
Favipiravir	Small molecule, antiviral, nucleoside analog	None	RCT	Not supported: RCTs do not show significant improvements for individuals taking this treatment, good safety profile
Remdesivir	Small molecule, antiviral, adenosine analog	Approved for COVID-19 (and EUA for combination with baricitinib)	RCT	Conflicting evidence from large WHO-led Solidarity trial vs US-focused RCT and other studies
N3	Small molecule, protease inhibitor	None	Computational prediction, *in vitro* studies	Unknown
ARBs & ACEIs	Small molecule, broad spectrum	None	Observational studies and some RCTs	Not supported: Observational study retracted, RCTs suggest no association
HCQ/CQ	Small molecule, broad spectrum	None	RCT	Not supported, possibly harmful: Non-blinded RCTs showed no improvement over SOC, safety profile may be problematic
Dexamethasone	Small molecule, broad spectrum	Used off-label	RCT	Supported: RCT shows improved outcomes over SOC, especially in severe cases such as CRS
Tocilizumab	Biologic, monoclonal antibody	Approved for CRS resulting from CAR-T therapy	RCT	Mixed results from RCTs: It appears that TCZ may work well in combination with dexamethasone in severe cases, but not as monotherapy
Casirivimab and imdevimab	Biologic, monoclonal antibodies	EUA	RCT	Supported: Reduced viral load at interim analysis
Bamlanivimab and etesevimab	Biologic, monoclonal antibodies	EUA	RCT	Supported: Phase 2 clinical trial showed reduction in viral load
SNG001	Biologic, interferon	None	RCT	Mixed results: support from initial RCT but no effect found in WHO’s Solidarity trial

## Importance

The COVID-19 pandemic is a rapidly evolving crisis. With the worldwide scientific community shifting focus onto the SARS-CoV-2 virus and the disease it causes, a large number of possible pharmaceutical approaches for treatment and prevention have been proposed. What is known about each of these potential interventions evolved rapidly throughout 2020 and early 2021. In March 2020, we began monitoring a range of candidates and have continued to update this manuscript as new information has become available. Some therapeutics have been supported, others have been revealed to be unlikely to confer any therapeutic benefits, and most require more data before a conclusion can be drawn. This rapidly changing area of research provides important insight into how the ongoing pandemic can be managed and also demonstrates the power of interdisciplinary collaboration to rapidly understand a virus and match its characteristics with existing or novel pharmaceuticals.
